# Capacitation of human naïve pluripotent stem cells for multi-lineage differentiation

**DOI:** 10.1242/dev.172916

**Published:** 2019-04-03

**Authors:** Maria Rostovskaya, Giuliano G. Stirparo, Austin Smith

**Affiliations:** Wellcome-MRC Cambridge Stem Cell Institute, Cambridge CB2 1QR, United Kingdom

**Keywords:** Competence, Differentiation, Epiblast, Human embryo, Lineage specification, Pluripotent stem cell

## Abstract

Human naïve pluripotent stem cells (PSCs) share features with the pre-implantation epiblast. They therefore provide an unmatched opportunity for characterising the developmental programme of pluripotency in *Homo sapiens*. Here, we confirm that naïve PSCs do not respond directly to germ layer induction, but must first acquire competence. Capacitation for multi-lineage differentiation occurs without exogenous growth factor stimulation and is facilitated by inhibition of Wnt signalling. Whole-transcriptome profiling during this formative transition highlights dynamic changes in gene expression, which affect many cellular properties including metabolism and epithelial features. Notably, naïve pluripotency factors are exchanged for postimplantation factors, but competent cells remain devoid of lineage-specific transcription. The gradual pace of transition for human naïve PSCs is consistent with the timespan of primate development from blastocyst to gastrulation. Transcriptome trajectory during *in vitro* capacitation of human naïve cells tracks the progression of the epiblast during embryogenesis in *Macaca fascicularis*, but shows greater divergence from mouse development. Thus, the formative transition of naïve PSCs in a simple culture system may recapitulate essential and specific features of pluripotency dynamics during an inaccessible period of human embryogenesis.

## Abstract (precis)

**Highlighted Article:** Human naïve pluripotent stem cells acquire competence for multilineage differentiation following a developmental trajectory that is comparable with the primate embryonic epiblast, and is facilitated by inhibition of the Wnt pathway.

## INTRODUCTION

Pluripotency refers to a flexible potential for individual cells to give rise to all lineages of the embryo. This property is a foundational feature in amniote embryogenesis (Sheng, 2015). Pluripotency extends from initial emergence of the epiblast a few days after fertilisation until lineage commitment during gastrulation. The period varies from ∼4 days in mouse and other rodents to 8-10 days or longer in primates, including *Homo sapiens*, and in many other mammals. Over this time, pluripotent cells change in character from the initial naïve condition to a lineage-primed state that is poised for commitment (Morgani et al., 2017; Nakamura et al., 2016; Nichols and Smith, 2009; Smith, 2017). This dynamic transition is manifest at the cellular level by epithelialisation and molecularly by reconfiguration of transcriptome, epigenome and metabolism (Bedzhov and Zernicka-Goetz, 2014; Buecker et al., 2014; Kalkan et al., 2017; Mohammed et al., 2017; Zhou et al., 2012).

Cultures of pluripotent stem cells (PSCs) can be derived from embryos (Evans and Kaufman, 1981; Martin, 1981; Thomson et al., 1998) or generated by molecular reprogramming (Takahashi and Yamanaka, 2006). Different PSC phenotypes present a spectrum of pluripotent states (Enver et al., 2009; Hackett et al., 2017; Hackett and Surani, 2014; Hough et al., 2014; Nichols and Smith, 2009), some of which show correspondence with stages of *in vivo* progression of the epiblast, whereas others may be artefactual products of the culture environment (Gokhale et al., 2015; Smith, 2013). In the mouse, canonical embryonic stem (ES) cells that are cultured in defined conditions are considered to be counterparts of the naïve epiblast from which they are derived (Boroviak et al., 2014; Brook and Gardner, 1997). By contrast, mouse postimplantation epiblast-derived stem cells (EpiSCs) (Brons et al., 2007; Tesar, 2005) resemble the gastrulating epiblast of the anterior primitive streak (Kojima et al., 2014; Tsakiridis et al., 2014) and are accordingly classified as primed (Nichols and Smith, 2009). Human and other primate PSCs, as conventionally established and propagated, are overtly different from mouse ES cells and are transcriptionally distinct from the pre-implantation epiblast (Nakamura et al., 2016; Rossant, 2015; Rossant and Tam, 2017; Yan et al., 2013). They display postimplantation features (Nakamura et al., 2016), although positioning on the developmental axis is uncertain, both because of variation between cell lines and culture conditions, and because there is no human reference available for early postimplantation embryogenesis. Recently, culture conditions have been devised that sustain human PSCs (hPSCs) with many of the expected properties of naïve pluripotency (Takashima et al., 2014; Theunissen et al., 2016, 2014). Naïve cells can be generated by resetting conventional PSCs (Guo et al., 2017), by somatic cell reprogramming (Kilens et al., 2018; Liu et al., 2017) or by derivation directly from dissociated human inner cell mass (ICM) cells (Guo et al., 2016). They exhibit transcriptome correlation with the pre-implantation epiblast (Nakamura et al., 2016; Stirparo et al., 2018) and show protein expression of naïve epiblast-specific transcription factors such as KLF4, KLF17 and TFCP2L1 (Guo et al., 2016; Takashima et al., 2014).

Human naïve PSCs provide an opportunity for simulation of the developmental programme of human pluripotency before gastrulation. They may thereby open a window into events that occur during the second week of gestation that cannot be characterised or even observed in human embryos *in utero*. This is a period of major change that appears to be crucial for establishing differentiation competence (Rossant and Tam, 2017; Sheng, 2015; Smith, 2017). Notably, mouse naïve ES cells do not differentiate directly into germ cell or somatic lineages, but first transit through the peri- and early postimplantation phase of epiblast development (Hayashi et al., 2011; Kalkan et al., 2017; Mulas et al., 2017). During this formative transition, naïve cells are proposed to gain competence for lineage induction through a process of capacitation (Kalkan and Smith, 2014; Smith, 2017). In mouse ES cells, capacitation occurs within 24-48 h (Hayashi et al., 2011; Mulas et al., 2017), which reflects the rapid rate of peri-implantation development in rodents (Acampora et al., 2016). For primate naïve PSCs, the process may be expected to extend over several days, in line with slower embryogenesis (Nakamura et al., 2016; Smith, 2017). However, current methods for capacitating naïve PSCs require prolonged culture for more than 20 days to achieve robust multilineage differentiation (Guo et al., 2017). The developmental relevance of this protracted conversion is further questioned by poor viability, cellular heterogeneity and rather low efficiency. Here, we set out to determine conditions under which naïve PSCs may recapitulate *in utero* progression to late epiblast, fully competent for germ layer induction.

## RESULTS

### Naïve hPSCs do not respond immediately to somatic lineage induction

**Fig. 1.**
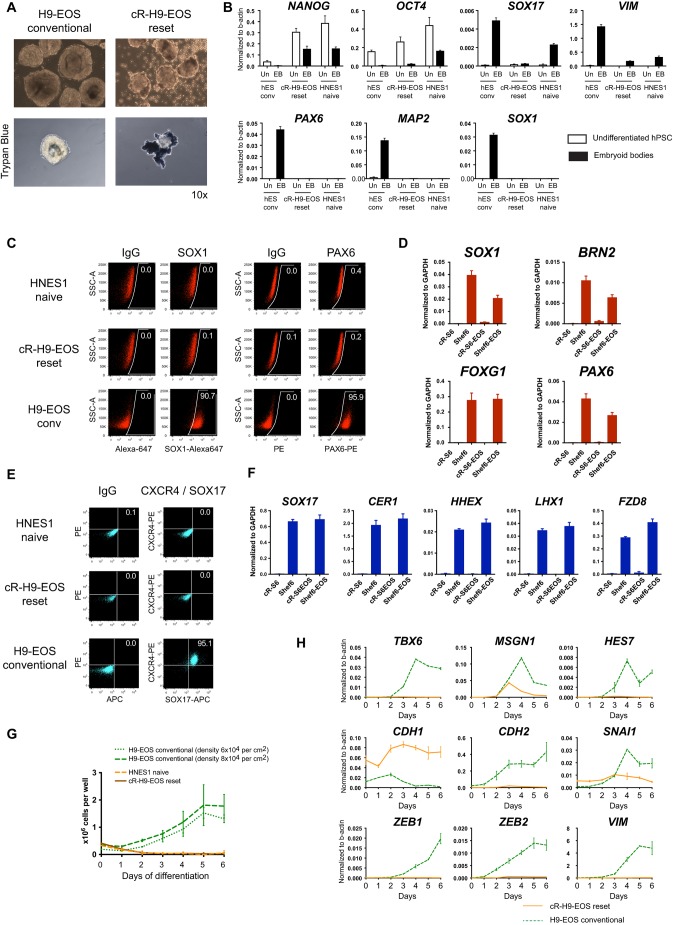
**Naïve hPSCs do not respond to lineage** **differentiation cues.** (A) Bright-field images of aggregates cultured for 7 days in N2B27, unstained and stained with Trypan Blue. Images were acquired with the same 10× objective and are shown in scale. (B) RT-qPCR analysis of marker expression after 14 days of aggregation culture. Here, and in all qPCR measurements, error bars represent s.d. of technical duplicates. (C) Flow cytometry analysis of intracellular marker staining after application of dual SMAD inhibition for neuroectoderm induction. SSC-A, forward scatter. (D) RT-qPCR analysis following dual SMAD inhibition. (E) Flow cytometry analysis of cell surface (CXCR4) and intracellular (SOX17) marker expression after application of definitive endoderm induction conditions. (F) RT-qPCR analysis in definitive endoderm induction conditions. (G) Cell counts in paraxial mesoderm induction conditions. Error bars are derived from two independent experiments. (H) RT-qPCR analysis following application of paraxial mesoderm induction protocol. EB, embryoid bodies; Un, undifferentiated.

Throughout this study we compared the conventional human ES (hES) cell line H9EOS with reset naïve derivative cR-H9EOS (Guo et al., 2017) and with the embryo-derived naïve line HNES1 (Guo et al., 2016). We first tested multilineage differentiation via embryoid body formation in non-instructive serum-free conditions, a context that is permissive for the three primary germ layers. PSCs were aggregated in suspension in N2B27 medium for up to 14 days. Conventional cells developed into typical embryoid body structures, with downregulation of pluripotency markers *NANOG* and *OCT4* (*POU5F1*), and robust expression of markers of the three germ layers (Fig. 1A,B). Naïve cells formed fewer and smaller aggregates, with extensive cell death. They retained substantial expression of *OCT4* and *NANOG*, whereas *SOX17* and *VIM* differentiation markers were modestly upregulated, but markers for neuroectoderm, *SOX1*, *PAX6* and *MAP2*, were not detected. The neural markers were also not expressed in outgrowths from naïve PSC aggregates (Fig. S1A). Thus, neural induction from naïve cells is not observed in embryoid body conditions, whereas other differentiation markers are expressed only at low levels.

Embryoid body differentiation is dependent on efficiency of cell aggregation and cell-cell interactions, parameters that are difficult to standardise. We therefore investigated the response of naïve cells to directed differentiation in adherent culture using protocols that have been proven for conventional PSCs. For these experiments, naïve PSCs were exchanged directly from self-renewal medium that contained inhibitors of MEK/ERK and aPKC (Guo et al., 2017; Takashima et al., 2014) to the respective lineage induction media.

For neuroectoderm induction, we employed dual SMAD inhibition (Chambers et al., 2009). By 10 days, quantification of SOX1 and PAX6 immunostaining by flow cytometry showed that H9EOS cultures comprise 90% neural lineage cells. In contrast, cR-H9EOS or HNES1 cultures contained fewer than 0.4% cells stained for either marker (Fig. 1C). Failure of direct neural induction is consistent with our previous observations on cR-Shef6 (Guo et al., 2017) and is further substantiated by the absence of mRNA expression for *SOX1*, *PAX6*, *BRN2* (*POU3F2*) and *FOXG1* (Fig. 1D). Definitive endoderm induction (Loh et al., 2014) applied to conventional hPSCs such as H9EOS or Shef6 generally results in ∼90% CXCR4+ SOX17+ cells detected by flow cytometry on day 3. In contrast, naïve PSC cultures remained negative for both markers (Fig. 1E), which was again consistent with previous observations (Guo et al., 2017). Naïve PSCs also failed to upregulate mRNA for *SOX17*, *CER1*, *HHEX*, *LHX1* and *FZD8* (Fig. 1F). During paraxial mesoderm differentiation (Chal et al., 2016), conventional hPSCs expanded during the 6-day protocol (Fig. 1G), underwent epithelial-to-mesenchymal transition (EMT), upregulated markers that are characteristic for paraxial mesoderm and EMT (*TBX6*, *MSGN1*, *HES7*, *CDH2*, *SNAI1*, *ZEB1*, *ZEB2* and *VIM*) and downregulated epithelial *CDH1* (Fig. 1H). In contrast, naïve PSCs showed high levels of cell death and the few remaining cells did not adopt mesenchymal morphology, lacked EMT markers, retained expression of *CDH1* and showed no or little upregulation of PM markers (Fig. 1G,H).

We further assessed the fate of naïve PSCs that were exposed to differentiation conditions, either via embryoid body formation (Fig. S1B) or by monolayer induction of neuroectoderm or definitive endoderm (Fig. S1C). Naïve and general pluripotency markers (*KLF4*, *KLF17*, *TFCP2L1*, *OCT4* and *NANOG*) were downregulated in most cases, although still detectable. Genes characteristic of postimplantation epiblast such as *TCF15*, *SOX11* and *HES1* (Boroviak et al., 2015; Nakamura et al., 2016) were generally upregulated, although to variable levels. These observations indicate that upon withdrawal from self-renewing conditions a proportion of naïve PSC may progress towards a postimplantation formative epiblast identity irrespective of environment.

These findings confirm and extend previous indications (Guo et al., 2017; Liu et al., 2017) that human naïve PSC lack competence to respond productively to inductive cues for lineage specification.

### Naïve hPSCs begin transition following withdrawal from self-renewal culture

We have previously shown that naïve hPSCs are able to differentiate into somatic lineages following a period of adaptation to culture in conventional hPSCs media, such as mTESR, FGF/KSR or E8 (Guo et al., 2017; Takashima et al., 2014). Therefore, we surmise that naïve hPSCs can be capacitated for somatic lineage induction, a process that we have termed formative transition (Smith, 2017). However, this transition is accompanied by significant cell death and considerable cellular heterogeneity in the above conditions; moreover, it takes longer than 20 days before stabilisation in a conventional PSC-like state (Guo et al., 2017). We therefore sought to achieve capacitation with improved consistency and efficiency, and over a developmentally more relevant time scale.

**Fig. 2.**
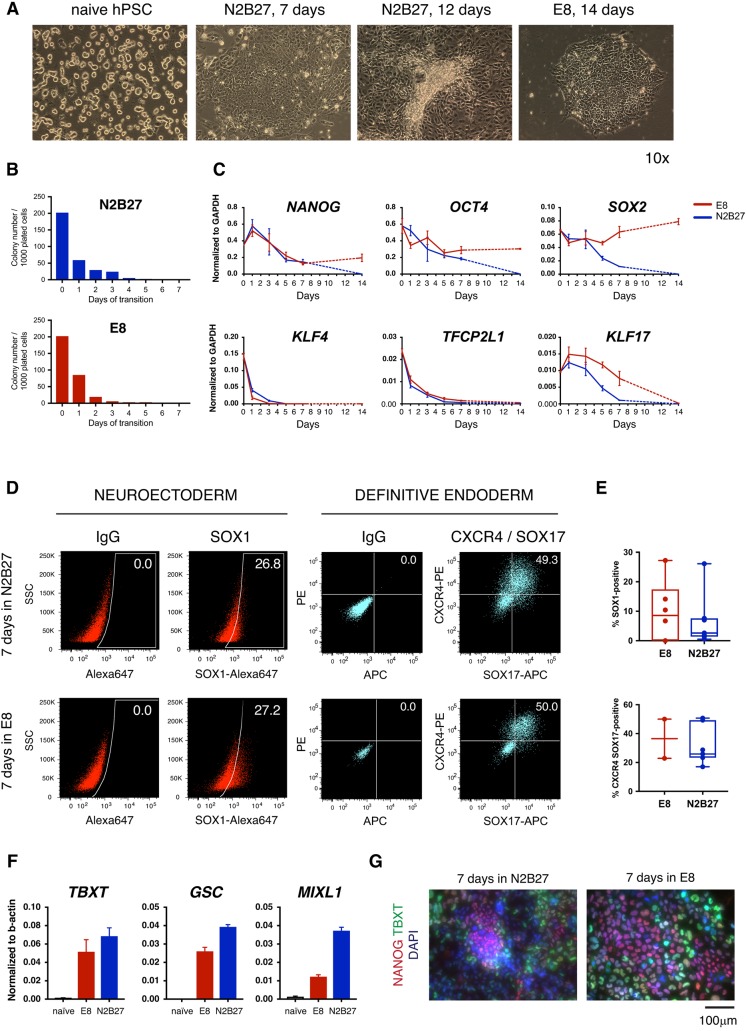
**Transition of naive hPSCs in N2B27 only.** (A) Bright-field images of naïve hPSCs and cultures in N2B27 for 7 or 12 days, or in E8 for 14 days. Images were acquired with the same 10× objective and are shown in scale. (B) Colony counts after culture in N2B27 or E8 and replating at low density in naïve PSC conditions. (C) RT-qPCR analysis of pluripotency marker expression during culture in N2B27 or E8. Dashed lines indicate that the intermediate time points were not analysed. (D) Flow cytometry analysis of neuroectoderm and definitive endoderm markers following induction after 7 days in N2B27 or E8. SSC, side scatter. (E) Summary of efficiencies of differentiation after 7 days in N2B27 or E8, from multiple independent experiments. (F) RT-qPCR analysis of meso-endodermal marker expression after 7 days in N2B27 or E8. (G) Immunostaining for TBXT and NANOG on day 7 in the indicated conditions.

We first compared capacitation in E8, which contains TGFβ and FGF2 (Chen et al., 2011), with transition in N2B27 without added growth factors. In both conditions, a proportion of cells acquired and maintained flattened epithelioid morphology, similar to conventional hPSCs, during the first 7 days. However, we observed that cell survival was much improved in N2B27. In E8, cells could be propagated further, albeit with considerable cell death, and eventually stabilise in a conventional PSC-like state. In contrast, in N2B27 the population became increasingly heterogeneous after day 7, and by day 12 all cells appeared to be differentiated and had ceased proliferation (Fig. 2A).

We tested the ability to re-form naïve colonies after periods in E8 or N2B27. Cells were re-plated at clonal density in naïve PSC culture conditions each day after switching to E8 or N2B27, and alkaline phosphatase-positive colonies were scored 5-7 days later. In both media, the proportion of cells that were capable of colony formation dropped markedly during the first 3 days (Fig. 2B). We monitored expression of pluripotency genes by RT-qPCR (Fig. 2C). In both media, naïve-specific transcription factors *KLF4* and *TFCP2L1* were downregulated over 3 days, whereas *KLF17* reduced more gradually. *OCT4* and *NANOG* declined less and then stabilised from day 7 in E8 but continued to fall in N2B27. *SOX2* was relatively stable in E8 but downregulated progressively in N2B27.

We assayed monolayer differentiation into neuroectoderm and definitive endoderm after 7 days of treatment, when cells in N2B27 or E8 appear to be similar. Both populations produced SOX1-positive neuroectodermal cells and CXCR4/SOX17 double positive endoderm (Fig. 2D). However, the efficiencies of differentiation from multiple independent experiments were variable and generally low, with maximum values ∼27% for neuroectoderm and ∼50% for definitive endoderm (Fig. 2E). In contrast, we have previously shown that longer-term culture (>20 days) in E8 results in efficiencies of 80-90% for both lineages, which is comparable with conventional PSCs (Guo et al., 2017).

These results indicate that naïve cells can reach somatic lineage competence over 7 days, with or without provision of exogenous FGF and TGFβ, but these conditions are only partially effective.

### Inhibition of Wnt signalling facilitates capacitation

**Fig. 3.**
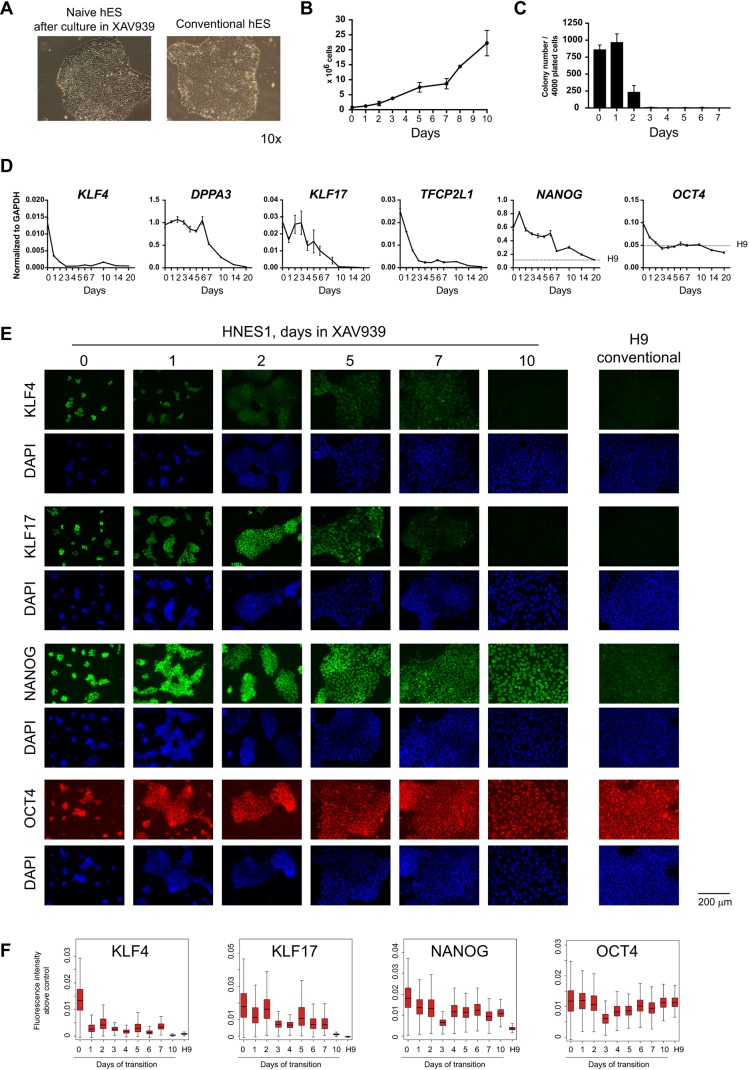
**Transition of naïve hPSCs is facilitated by XAV939.** (A) Bright-field images of conventional hPSCs and naive hPSCs before and after culture in N2B27 plus XAV939 for 14 days. Images were acquired with the same 10× objective and are shown in scale. (B) Cell counts during transition in XAV939; error bars represent s.d. from three independent experiments. (C) Colony counts after culture in XAV939 and replating at low density in naïve PSC conditions. (D) RT-qPCR assay of pluripotency gene expression during transition in XAV939. Dotted lines indicate expression in conventional H9 hES cells. (E) Immunofluorescent staining for pluripotency markers during transition in XAV939. H9 conventional hPSCs provide reference staining. (F) Quantification of intensity of immunostaining. Mean intensity of cells stained with the secondary antibodies only was subtracted from the measurement of each cell that was stained with specific antibodies. The resulting values of intensity above control are represented as boxplots.

At day 7 we noted a significant fraction of *TBXT* (brachyury)-expressing cells that were exclusive to NANOG-positive cells (Fig. 2F,G). *TBXT* is a known target of canonical Wnt signalling (Yamaguchi et al., 1999). We also detected the expression of Wnt ligands and pathway components (Fig. S2). We therefore examined whether endogenous Wnt activity might disrupt or divert the transition process. We tested capacitation in N2B27 supplemented with the tankyrase inhibitor XAV939 (2 µm), which blocks canonical Wnt signalling (Huang et al., 2009). In contrast to cultures in N2B27 alone or E8, we observed a relatively uniform establishment of epithelial morphology that was similar to conventional hPSCs throughout the culture in the presence of XAV939 (Fig. 3A). Cells expanded continuously throughout this conversion (Fig. 3B). Moreover, unlike in N2B27 only, proliferation was sustained for at least 20 days. Thereafter, the cultures could still be maintained, but became heterogeneous. We found that addition of FGF2 and activin A to XAV939 (hereafter XAF) after day 10 allowed for continued propagation with minimal overt differentiation for at least 50 days (Fig. S3). XAF is similar to the medium that is used for culturing mouse EpiSC as more homogeneous populations (Sumi et al., 2013; Tsakiridis et al., 2014).

A colony assay showed that the ability to self-renew in naïve conditions was greatly diminished after 2 days in N2B27 plus XAV and almost eliminated by day 3, similar to cultures in N2B27 only or E8 (Fig. 3C). Likewise, naïve pluripotency factor (*KLF4*, *TFCP2L1*) transcripts were reduced to a very low or undetectable level over this period, whereas *KLF17* declined more slowly and was extinguished only by 10 days of transition. In distinction to cultures in N2B27 alone, however, *NANOG* and *OCT4* expression stabilised from day 7 at similar levels to those observed in conventional hPSCs (Fig. 3D). We also noted that *DPPA3* was downregulated from day 6 onwards. These observations were substantiated by immunostaining (Fig. 3E,F).

**Fig. 4.**
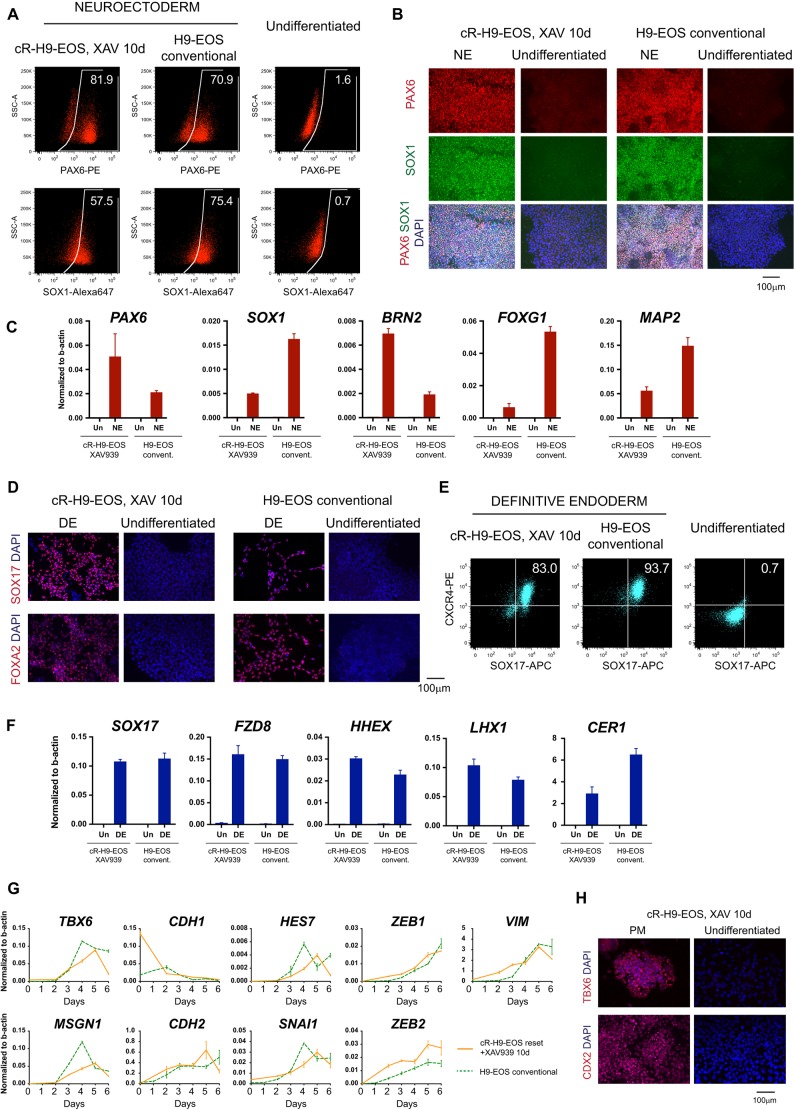
**Differentiation competence of hPSCs after formative transition in XAV939.** (A-C) Neuroectoderm induction after transition in XAV939, parallel treatment of conventional hPSCs and naïve hPSCs after capacitation. Neuroectoderm markers were examined using flow cytometry (A), immunostaining (B) and RT-qPCR (C). (D-F) Definitive endoderm induction after transition in XAV939. Markers were examined using immunostaining (D), flow cytometry (E) and RT-qPCR (F). (G,H) Differentiation to paraxial mesoderm after transition in XAV939. Markers were analysed using RT-qPCR (G) and immunostaining for TBX6 and CDX2 (H). DE, definitive endoderm; NE, neuroectoderm; PM, paraxial mesoderm; SSC-A, side scatter; Un, undifferentiated (conventional and capacitated hPSCs that were not induced to differentiation).

We tested directed differentiation after 10 days in XAV939 when cells displayed a pluripotency marker profile that was similar to conventional PSCs. Upon dual SMAD inhibition, we observed robust upregulation of PAX6 and SOX1, as shown by immunostaining and quantified by flow cytometry (Fig. 4A,B). The efficiency was comparable with conventional hPSCs. RT-qPCR confirmed expression of these markers, along with *BRN2*, *MAP2* and *FOXG1* (Fig. 4C). These cells could be further differentiated to post-mitotic neurons, which was validated by immunostaining for TUBB3 (β-III-tubulin), MAP2 and NEUN (RBFOX3), and by RT-qPCR for *MAP2*, *NEUN*, *NCAM1* and *ASCL1* (Fig. S4A,B). Definitive endoderm induction was also highly efficient, and was assayed by immunostaining for SOX17 and FOXA2 (Fig. 4D), and by flow cytometry which quantified co-expression of CXCR4 and SOX17 in 83.0% of cells (Fig. 4E). Importantly, the primitive endoderm marker PDGFRa (Blakeley et al., 2015; Plusa et al., 2008; Stirparo et al., 2018; Yan et al., 2013) was not induced (Fig. S4C). RT-qPCR showed the expression of definitive endoderm markers at levels that were similar to induction from conventional hPSC (Fig. 4F). Differentiation could be continued to PDX1-expressing foregut progenitors, with efficiencies >80% (Fig. S4D). Finally, in response to paraxial mesoderm induction, lineage markers and EMT genes were upregulated whereas *CDH1* was downregulated (Fig. 4G). TBX6 and CDX2 protein expression was confirmed by immunostaining (Fig. 4H). Paraxial mesoderm identity was substantiated by further differentiation to myotubes with expression of transcripts for sarcomeric proteins TTN and DMD (Fig. S4E). Immunostaining for sarcomeric myosin showed striated myofibers (Fig. S4F) and spontaneous contractions confirmed functional sarcomere assembly (Movie 1).

We repeated capacitation with XAV939 on multiple naïve PSCs, including: embryo-derived HNES1, HNES5c1 and HNES5c5; reset ES cells, cR-H9EOS and cR-S6EOS; reset induced PSCs (iPSCs), cR-LQT1. Lineage competence was consistently achieved across multiple independent experiments (Fig. S5 for selected cell lines; Table S1).

XAV939 is a potent tankyrase (TNKS and TNKS2) inhibitor (Huang et al., 2009), which stabilises the axin-GSK3β complex resulting in degradation of β-catenin, but potentially can affect other cellular pathways (Lehtiö et al., 2013). In order to confirm that capacitation is facilitated specifically by inhibition of the Wnt pathway, we tested an alternative mode of inhibition. IWP2 and C59 act on porcupine O-acyltransferase to prevent Wnt processing and secretion (Chen et al., 2009; Proffitt et al., 2013). Naïve hPSCs that were cultured in N2B27 with C59 or IWP2 also produced cells with flat epithelioid morphology (Fig. S6A) that displayed similar multi-lineage competence as cells that were cultured with XAV939 (Fig. S6B-F).

PSCs that were capacitated in XAV939 for 10 days could be further propagated for multiple passages in either XAF or E8 without signs of growth arrest or differentiation. In either media, cells maintained their abilities to produce derivatives of three germ layers with efficiencies similar to conventional PSCs, even >33d after initiation of transition (Fig. S7)

From these results, we conclude that Wnt inhibition facilitates formative transition of human naïve cells. By 10 days, cells appear to be fully capacitated to produce neuroectoderm, endoderm and mesoderm lineage precursors that can undergo further differentiation into tissue progenitors and post-mitotic cell types.

### Global gene expression profiling during capacitation of naïve hPSCs

To characterise gene expression dynamics during capacitation, we performed whole transcriptome RNA sequencing at days 0, 1, 2, 3, 7 and 10 (Fig. S8A). In addition, on day 10 cells were split into two conditions for continued maintenance, E8 or XAF medium, and passaged until 22-28 days in total (indicated as d22+). Conventional H9EOS hES cells in E8 were used as a reference. We prepared biological triplicate samples for all cell lines and conditions.

Pearson correlation analysis based on all expressed genes divided samples into two major populations: early, day 0-3, and late, day 7 onwards (Fig. S8B). HNES1 and cR-H9EOS samples were highly correlated over the entire time course, which indicates a shared trajectory. We determined the number of variable genes for all pairwise comparisons, which confirmed that differential expression increased markedly between early and late samples (Fig. S8C).

We further examined expression dynamics after 10 days, following transfer to either E8 or XAF. Pearson correlation analysis of samples on day 10, day 22+ and control cells, reveals overall similarity, with correlation coefficients greater than 0.95 in all comparisons (Fig. S8D). Principal component analysis (PCA) shows two major groups that correlate to the culture conditions; E8 medium or XAV939-containing media (Fig. S8E). Of note, cR-H9EOS cells that were capacitated and then maintained in E8 medium are most similar to conventional H9EOS hES cells that were cultured in E8. Thus, following capacitation, naïve cells can regain their original conventional PSC state in terms of global gene expression if they are expanded in comparable culture conditions.

**Fig. 5.**
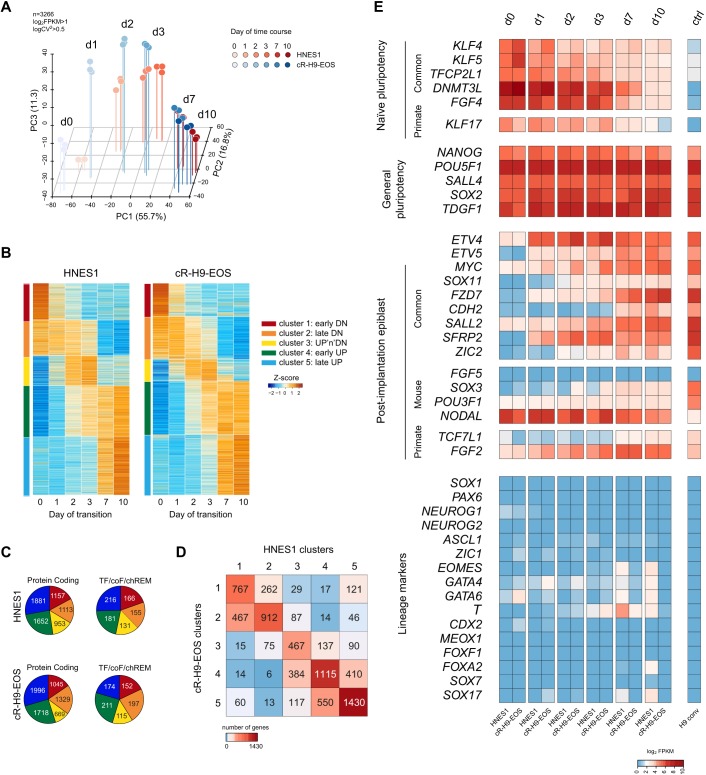
**Global transcriptome analysis during formative transition.** (A) PCA of HNES1 and cR-H9EOS during formative transition. For each cell line, three independent experiments are represented. (B) Heat maps showing expression of variable genes assigned to five dynamic clusters, derived separately for HNES1 and cR-H9-EOS. (C) Numbers of protein coding genes, and of transcription factors, co-factors and epigenetic remodellers (TF/coF/REM), within the five dynamic clusters. (D) Comparison of gene content of clusters between HNES1 and cR-H9-EOS; 61.7% of genes belong to the same clusters and 83.9% belong to the same or similar clusters. (E) Heat map of RNAseq expression values for selected genes during formative transition.

We examined the dynamics of gene expression during the time course. PCA for all variable genes confirmed that HNES1 and cR-H9EOS follow similar trajectories (Fig. 5A). Biological triplicates showed high consistency. We then generated a list that comprised all genes that are differentially expressed between any two time points of the time course and in at least one cell line (padj<0.01; fold change>2). Soft clustering analysis distinguished five major clusters, which were identified by minimising the total variation within each cluster (Fig. S9A): 1, early down; 2, late down; 3, up and down (up-n-dn); 4, early up; 5, late up (Fig. 5B,C). We compared the clusters between the two cell lines, considering genes of the same and similar clusters. Similar clusters are those in which genes changed expression in the same direction, but with different dynamics: clusters 1 and 2 (downregulated); clusters 4 and 5 (upregulated). For the two cell lines, 61.7% of genes belonged to the same clusters, and 83.9% were in the same and similar clusters (Fig. 5D,E). PCA showed proximity between the same clusters, indicating that not only the dynamics, but also the levels of gene expression are similar between the two cell lines (Fig. S9B).

We inspected the representation of KEGG pathways that are related to the clusters (Fig. S9C). We noted that genes that are related to oxidative phosphorylation are mostly downregulated (clusters 1 and 2), whereas those that are associated with glycolysis show no prevailing direction of change (Fig. S10A, Table S6). We used TMRE staining to assay mitochondrial membrane potential, and observed reduced activity in capacitated compared with naïve PSCs (Fig. S10B-D). This is in accord with the reduction of mitochondrial respiration that is observed during formative transition in mouse (Fiorenzano et al., 2016; Kalkan et al., 2017; Zhou et al., 2012). Genes that are related to focal adhesion, cell-cell adhesion, ECM-receptor interactions and adherent junctions were predominant in the upregulated clusters (4 and 5). We inspected the expression of a panel of genes that are associated with tight junctions, cell polarity, integrins and cadherins, and observed that many are upregulated, some continuously but others only after day 3 or even later (Fig. S11). An exception is *Cdh1* (E-cadherin), which is downregulated but still expressed. These data highlight the gain of epithelial features as a major aspect of formative transition *in vitro*, consistent with the development of a laminar epithelial epiblast disk in the early postimplantation embryo (Sheng, 2015).

We inspected the expression of ligands and receptors of the TGFβ family and noted that both nodal and the convertase furin are downregulated at the end of transition, whereas TGFBR1, TGFBR2 and ACVR2B receptors gain expression (Fig. S12). These dynamics are consistent with the requirement for exogenous activin for robust expansion after capacitation.

Dynamics of selected genes were then assessed (Fig. 5E). Genes that are characteristic for the naïve epiblast, such as *KLF4*, *TFCP2L1*, *DNMT3L*, *FGF4* and *KLF17* (Stirparo et al., 2018; Takashima et al., 2014), were downregulated to very low levels. General pluripotency factors *NANOG* and *OCT4* were partially downregulated, whereas *SOX2* expression was relatively constant. These results are consistent with RT-qPCR and immunofluorescence analyses. We noted that DNMT3B was upregulated early whereas DNMT3A expression was maintained at a similar level (Fig. S12). Markers that are characteristic for the early postimplantation epiblast (Boroviak et al., 2015; Nakamura et al., 2016) were among the upregulated genes, including *SOX11*, *FZD7*, *CDH2* and *SALL2*. We also evaluated genes that differ in expression between mouse and primate postimplantation epiblast. Among the genes that were reported as specific for mouse early postimplantation stages (Boroviak et al., 2015), some were not upregulated (*FGF5*, *POU3F1*, *NODAL*), whereas others showed mild upregulation (*SOX3* and *SOX4*). On the other hand, markers that are distinctive for primate postimplantation epiblast were upregulated, among them *TCF7L1* (which encodes TCF3) and *FGF2*. Of note, most early lineage markers, such as *SOX1*, *PAX6*, *CDX2*, *GATA4*, *GATA6*, *SOX17* and *FOXA2* showed very low or undetectable expression, even at the end of the time course. Only *TBXT* and *MIXL1* were found at low but detectable levels. Furthermore, this profile was maintained after extended expansion in E8 or XAF (Fig. S13). We validated the dynamics of gene expression during formative transition using RT-qPCR analysis of independent experiments using HNES1, HNES5c2 and cR-H9EOS cell lines (Fig. S14). This also revealed that, although cells at day 7 and 10 of capacitation share many features, some genes such as *KLF17*, *TCF7L1* and *CDH2* show ongoing expression changes between these timepoints, which indicates that up to 10 days are required to achieve a stable profile.

These analyses reveal that there is a global and dynamic reconfiguration of gene expression during formative transition, with implications for metabolism, transcriptional regulation and cell biological properties.

### Comparison of gene expression dynamics between *in vitro* capacitation and *in utero* progression of primate epiblast

**Fig. 6.**
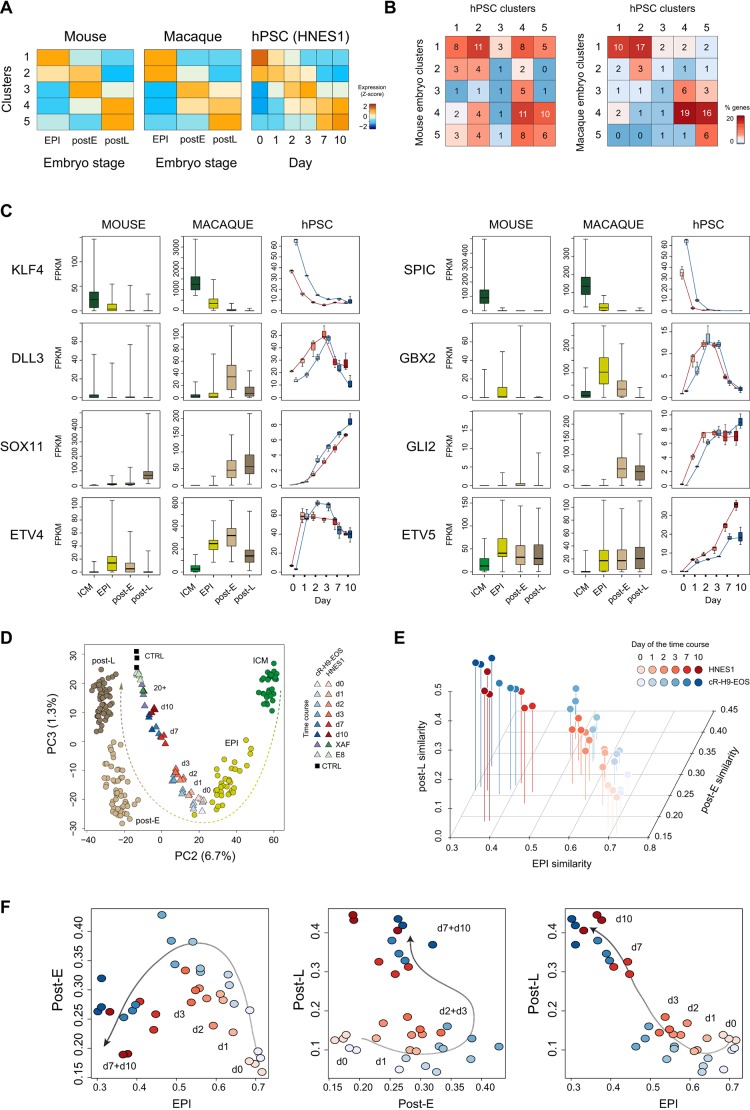
**Comparison of gene expression during formative transition of hPSCs *in vitro* and *M. fascicularis* embryonic epiblast *in utero*.** (A) Soft clustering analysis of variable genes during developmental progression of mouse and macaque epiblast *in utero*, and transition of hPSCs *in vitro*; average level of gene expression per cluster is indicated. (B) Comparison of clusters of variable genes between progression of mouse or macaque epiblast *in utero* and hPSCs *in vitro*. (C) Expression of selected genes during progression of mouse and macaque epiblast *in utero* and hPSCs *in vitro*. (D) PCA of macaque epiblast single cells at different stages of development *in utero* and hPSC populations during formative transition *in vitro*. (E,F) Fractions of similarity of hPSCs during formative transition to embryonic stages EPI, post-E and post-L of the macaque embryo: 3D plot (E) and 2D projections (F).

To assess concordance between *in vitro* capacitation and formative transition *in vivo*, we compared our transcriptome results with the published single cell RNAseq data for early embryogenesis in the mouse (Boroviak et al., 2018; Mohammed et al., 2017) and the cynomolgus monkey, *Macaca fascicularis* (Nakamura et al., 2016). From the mouse embryo dataset, we used cells of the pre-implantation epiblast (day 4.5, EPI), and of the early (day 5.5, post-E) and late (day 6.5, post-L) postimplantation epiblast. From the macaque embryo, we selected cells that were assigned as preEPI, post-E and post-L epiblast in the original study. For both species, we selected variable genes during epiblast progression (padj<0.01, fold change >2) and identified five clusters of genes with distinct dynamic behaviour using soft clustering analysis (Fig. 6A).

We compared the dynamics of gene expression in the clusters of the embryonic epiblast progression with the *in vitro* time course. We noted that clusters 1, 2, 4 and 5 showed comparable behaviour between mouse and macaque embryo and hPSC, and could be regarded as equivalent. Cluster 3 genes behaved slightly differently. In mouse and macaque embryos, these genes were upregulated from EPI to post-E, then downregulated in post-L, but maintained at a higher level than in EPI. In contrast, most genes of cluster 3 of the *in vitro* time course showed similar expression at the start and end points. Therefore, we considered two groups of similar clusters: downregulated (embryo 1 and 2, and hPSC 1 and 2); upregulated (embryo 3, 4 and 5, and hPSC 4 and 5). Examination of the overlap between the clusters of dynamic genes during the hPSC transition and in the mouse embryo revealed that a significant proportion of genes belonged to the same (28.9%) or similar clusters (66.3%). Furthermore, comparison of the hPSC transition with the macaque embryo showed a greater overlap, 39.0% of variable genes were in the same clusters and 83.1% were in similar clusters (Fig. 6B).

Examples of genes with similar dynamics during capacitation of hPSC and in both mouse and macaque embryos are: *KLF4* and *SPIC*, downregulated; *GBX2*, up and down; *SOX11*, *ETV4* and *ETV5*, upregulated (Fig. 6C). Genes that were dynamically expressed in hPSCs and in the macaque that were not detected in the mouse and have not been characterised in the context of pluripotency progression include *DLL3* and *GLI2*. Overall, therefore, *in vitro* capacitation of hPSC shares gene expression features with mouse, but more closely resembles development of the primate epiblast.

We further focused on the comparison of the formative transition of hPSCs *in vitro* and the macaque embryo epiblast *in utero*. We performed PCA with hPSCs during the transition time course and the embryo cells from ICM, EPI, post-E and post-L stages. The two datasets are separated in PC1, which can be attributed to differences in methodology (single cell versus bulk RNAseq, alternative-sequencing chemistries), species and environment. PC2 and PC3 reflect gradual progression in the embryo from ICM to EPI, then to post-E and finally to post-L (Fig. 6D). Naïve hPSCs are most similar to the EPI, which is consistent with previous analyses (Nakamura et al., 2016; Stirparo et al., 2018), and during *in vitro* capacitation hPSCs align with the progression of the embryonic epiblast. We validated these results by comparing samples taken during capacitation with samples taken from the embryo stages EPI, post-E and post-L (Fig. 6E,F). Gene expression for the embryo samples was calculated as average values of single cells. Using quadratic programming (Gong and Szustakowski, 2013), we measured relative similarities of hPSC samples to EPI, post-E and post-L. Similarity to EPI decreased continuously during capacitation. The fraction of similarity to post-E increased during the early steps of the time course (days 1-3) and then reduced on days 7-10. Conversely, similarity to post-L remained relatively low during days 0-3, and increased on days 7 and 10. Therefore, the trajectory of *in vitro* capacitation follows the progression of the embryonic epiblast of primate embryos *in utero*.

## DISCUSSION

Our results demonstrate that human naïve PSCs are not equipped to enter directly into lineage specification but must first undergo a formative transition. During this capacitation process, PSCs downregulate naïve pluripotency transcription factors, rewire metabolism and signalling pathways, develop epithelial character and become fully competent for differentiation to embryonic lineages. At the end of this transition, cells exhibit dependence on exogenous FGF and activin/TGFβ for continued expansion. Capacitation takes up to 10 days and follows a trajectory and timeline that is reflective of the progression of the primate epiblast from ICM to gastrulation. These findings are consistent with the postulate of naïve pluripotency as a *tabula rasa*, in which potential for multi-lineage differentiation is created, but not actuated (Nichols and Smith, 2009; Smith, 2017).

Classically, pluripotency is considered the capacity of single cells to form all embryonic lineages with no predetermination. Highlighting capacitation as a pre-requisite for multi-lineage differentiation accords with a more refined concept, in which pluripotency refers to potential rather than actual capacity. Naïve pluripotent cells can ultimately give rise to all cell types of the body, but only via an obligate transition during which competence is instated. Until recently, this distinction has been obscured by the compression of events in rapidly advancing mouse ES cell differentiation. Indeed, mouse ES cells have often been presented as responding directly to lineage cues. However, closer inspection has revealed that, in reality, naïve cells first lose ES cell identity and transit to a population, which is termed epiblast-like (EpiLC) or formative, that is enabled for lineage induction (Buecker et al., 2014; Hayashi et al., 2011; Hoffman et al., 2013; Kalkan et al., 2017; Mulas et al., 2017). When human naïve PSCs are exposed directly to inductive environments, they show a heterogeneous response, which includes cell death, partial transition to postimplantation epiblast and differentiation into miscellaneous phenotypes. Acquisition of competence is, therefore, a shared requirement for mouse and human naïve PSCs.

For mouse ES cells, capacitation can proceed in medium without growth factors or inhibitors other than insulin. In human cells, however, this process is unreliable because of disruption by endogenous Wnt activity. Interestingly, and in contrast to mouse ES cells, human naïve PSC self-renewal may also be perturbed by Wnt signalling (Guo et al., 2017; Theunissen et al., 2016; Zimmerlin et al., 2016). Indeed, for most experiments in the present study we used human naïve PSCs that had been maintained in the presence of XAV939 along with MEK and aPKC inhibitors (Bredenkamp et al., 2019). Regardless of the naïve PSC maintenance condition, we found that, during capacitation, continuous Wnt pathway inhibition markedly improves the efficiency and consistency of transition. This finding may relate to the known role of Wnt signalling in promoting axis formation and gastrulation (Huelsken et al., 2000; Liu et al., 1999; Morkel et al., 2003). Activation of the Wnt pathway via GSK3 inhibition is also a key component in two of the three lineage induction protocols for hPSCs. Our findings indicate that, before competence, the transitional epiblast should be shielded from Wnt stimulation to avoid inappropriate gene induction and miscellaneous differentiation.

The formative transition is considerably faster in mice than in primates, both *in vitro* and in the embryo. One contribution to different developmental timing may be the appropriation of *TCF7L1* (TCF3) in the mouse but not in the primate naïve epiblast (Boroviak et al., 2018). In mouse ES cells, TCF3 acts as a potent repressor of naïve network transcription factors (Martello et al., 2012). Inhibition of glycogen synthase kinase 3 supports mouse ES cell self-renewal principally by abrogating the repressor activity of TCF3 (Wray et al., 2011) and TCF3 depletion substantially delays the exit of mouse ES cells from naïve pluripotency (Betschinger et al., 2013; Guo et al., 2011; Pereira et al., 2006). Without TCF3, therefore, human naïve cells lack the major accelerator of naïve state exit. Subsequent development of lineage competence is also slower in human, however, which implicates additional determinants of overall transition timing.

The slower pace of formative transition in humans may make the sequence of events easier to delineate and mechanistically dissect than in the mouse. Indeed, whereas exit from the naïve state and gain of multilineage competence are difficult to dissociate in mouse ES cells (Mulas et al., 2017), they appear to be separated by several days in the human system. Accordingly, we identified two major waves of dynamic transcriptome change. Over the first 3 days the cells downregulate a subset of naïve factors (KLF4, TFCP2L1), which is coincident with the reduction in ability to reform naïve colonies. Some postimplantation markers (TCF15, FGF2 and HES1) are upregulated early, and oxidative phosphorylation components are reduced. The end of this period marks the exit from naïve pluripotency for the bulk population. The second wave of transcriptional changes features the loss of other naïve factors (KLF17, DPPA3 and DPPA5), a marked upregulation of multiple genes that are associated with epithelial function, and the gain of TCF7L1 and TCF7L2, which are mediators in the canonical Wnt pathway. This complex profile indicates that the formative transition is wide reaching and comprised of distinct steps.

Gene expression dynamics during capacitation of naïve hPSCs exhibit similarity with epiblast progression in a non-human primate embryo, which indicates that the transition path is not merely an *in vitro* phenomenon. In the embryo, the epiblast undergoes profound cellular and molecular changes during the peri-implantation and early postimplantation period, in preparation for gastrulation (Acampora et al., 2016; Mohammed et al., 2017; Nakamura et al., 2016; Peng et al., 2016). Our transcriptome analyses indicate that hPSCs acquire full competence for somatic cell differentiation once they reach a state that is similar to the late pre-gastrulation epiblast. During subsequent expansion in FGF2 with TGFβ or activin A, cells exhibit close resemblance to conventional hPSCs, which is consistent with elaboration of a growth factor-driven stem cell phenotype.

In summary, when shielded from Wnt signalling but exposed to ERK1/2 and aPKC activity that are inhibited during self-renewal (Takashima et al., 2014), human naïve PSCs convert efficiently to a pluripotent condition that is empowered for specification and commitment. This competent state lacks overt transcriptional lineage priming, but can be reliably induced to undergo productive differentiation into endodermal, mesodermal and neuronal cell types. Furthermore, once acquired, multilineage competence can be stably maintained, similar to in conventional human PSCs. At the technical level, this simple and reliable transition system provides a platform for systematic evaluation of the differentiation propensity and consistency of human naïve PSCs and thereby for rigorous comparison with other types of hPSC culture. More fundamentally, the naïve PSC transition provides a window into a crucial phase of human embryogenesis that cannot be accessed *in vivo*. It will be of great interest to characterise the sequence of molecular events and the mechanisms that underlie the acquisition of embryonic lineage competence in this system.

## MATERIALS AND METHODS

### hPSC lines

Experiments were performed in parallel throughout on the embryo-derived naïve hPSC line HNES1 (Guo et al., 2016) and the reset naïve hPSC line cR-H9EOS (Guo et al., 2017). H9EOS cells, which are conventional H9 hES cells (Thomson et al., 1998) that carry the EOS reporter (Guo et al., 2017), were used as reference. Validation experiments were carried out on two clonal embryo-derived naïve hPSC lines (HNES5c1 and HNES5c2; unpublished), and on reset hES cells (described by Guo et al., 2017) or reset iPSCs (LQT1; Chen et al., 2017; Moretti et al., 2010) that were generated using the method of Guo et al. (2017). HNES cell lines were derived with informed consent under licence from the Human Embryology and Fertilisation Authority.

### hPSC maintenance

Cells were cultured throughout in a humidified incubator with 5% O_2_ and 7% CO_2_ at 37°C.

Naïve hPSCs were maintained on irradiated mouse embryonic fibroblast (MEF) feeder cells in N2B27 supplemented with 1 µM PD0325901, 10 ng/ml human LIF (produced in house), 2 µM Gö6983 (Tocris Bio-Techne, 2285), and either 0.3-1 µM CHIR99021 (Guo et al., 2016; Takashima et al., 2014) or 2 µM XAV939 (Tocris Bio-Techne, 3748) (Guo et al., 2017). Geltrex (Thermo Fisher Scientific, A1413302) was optionally added to the medium at 1 µl/ml during replating to aid attachment. Cells were passaged using TrypLE (Thermo Fisher Scientific, 12605028). ROCK inhibitor (10µM; Y-27632, 688000, Millipore) was added for 24 h after passaging. Conventional hPSCs were cultured in E8 medium (prepared in house according to Chen et al., 2011) on Geltrex pre-coated plates and passaged using 0.5 mM EDTA in PBS.

### Capacitation

Before capacitation, naïve hPSCs were passaged once without feeders in naïve PSC medium plus Geltrex at 1 µl/cm^2^. For the formative transition, cells were dissociated with TrypLE and plated to Geltrex-coated tissue culture plates at a seeding density of 1.6×10^4^/cm^2^ in medium for naïve hPSCs supplemented with 10 µM ROCK inhibitor. After 48 h, cells were washed with DMEM/F12 supplemented with 0.1% bovine serum albumin (BSA). Capacitation was then performed in the following conditions: E8 medium; N2B27 medium without supplementation; N2B27 supplemented with a Wnt inhibitor [2 µM XAV939 (Tocris Bio-Techne, 3748), 1 µM IWP2 (Tocris Bio-Techne, 3533) or 1 µM WNT-C59 (Tocris Bio-Techne, 5148)]. The medium was renewed every 1-2 days. Cells were passaged at a 1:2 ratio at confluency using TrypLE and10 µM ROCK inhibitor. Cells were replated for lineage induction after 10 days, unless otherwise specified.

For expansion after capacitation, cells were cultured in either E8, or in N2B27 supplemented with 2 µM XAV939, 3 ng/ml activin A and 10 ng/ml FGF2 (XAF medium). The cells could also be maintained in capacitation conditions (2 µM XAV939 in N2B27), but were prone to differentiation beyond 20 days. During expansion, cells were cultured on Geltrex pre-coated tissue culture plates and passaged by dissociation with either 0.5 mM EDTA or TrypLE. In the latter case, 10 µM ROCK inhibitor was added for 24 h after dissociation.

Cells could be frozen during capacitation in N2B27 with 10% DMSO. ROCK inhibitor (10 µM) was added for 24 h after thawing. Cells were passaged after thawing before setting up differentiation assays.

### Colony assay

For the colony formation assay, cells were dissociated with TrypLE and plated to Geltrex-coated 12-well plates at a density 1, 2 or 4×10^3^ cells/well in naïve medium supplemented with 10 µM ROCK inhibitor. After 5-7 days, colonies were fixed and stained for alkaline phosphatase (Sigma-Aldrich, 86R). Whole-well images were acquired using an Olympus IX51 inverted microscope and CellSens software and colonies were scored either manually or automatically using Ilastik and Fiji software.

### *In vitro* differentiation

#### Embryoid bodies

hPSCs were aggregated in Aggrewell plates with 400 µm microwells (Stemcell Technologies, 34411) in N2B27 medium supplemented with 10 µM ROCK inhibitor. After 2 days, aggregates were flushed from the wells with N2B27 medium and transferred to non-adhesive six-well plates for further culture in suspension. For outgrowth differentiation, on day 7 aggregates were plated on tissue culture grade plates coated with Geltrex. RT-qPCR analysis was performed on day 14.

#### Neuroectoderm

Neuroectoderm was induced based on Chambers et al. (2009) in N2B27 medium supplemented with 1µM A8301 (Tocris Bio-Techne, 2939) and 500 nM LDN193189 (alternative name DM3189, Axon Medchem, 1509), for 10 days. For subsequent neuronal differentiation, cells were dissociated after 10 days, passaged to plates that were pre-coated with poly-L-ornithine and laminin (both from Sigma-Aldrich) in N2B27 medium at a density of 10^5^ cells/cm^2^, and cultured for an additional 30 days.

#### Endoderm

Definitive endoderm was induced according to Loh et al. (2014). Cells were cultured in CDM2 basal medium that was supplemented with 100 ng/ml activin A (produced in house), 100 nM PI-103 (Tocris Bio-Techne, 2930), 3 µM CHIR99021, 10 ng/ml FGF2 (produced in house), 3 ng/ml BMP4 (PeproTech, 120-05ET), 10 µg/ml heparin (Sigma-Aldrich, H3149) for one day. For the next 2 days the following supplements were applied: 100 ng/ml activin A, 100 nM PI-103, 20 ng/ml FGF2, 250 nM LDN193189, 10 µg/ml heparin. Further induction of foregut progenitors was performed according to Rezania et al. (2014) with analysis at the S4 stage.

#### Paraxial mesoderm

Differentiation to paraxial mesoderm and myotubes was performed according to Chal et al. (2016). Cells were cultured in 3 µM CHIR99021 and 500 nM LDN193189 for 6 days, with addition of 20 ng/ml FGF2 from days 3-6. Multi-step induction of myotubes was continued up to 40 days of differentiation.

### Quantitative RT-PCR

Total RNA was extracted using Reliaprep RNA Miniprep (Promega) and 200-500 ng was used for reverse transcription using GoScript Reverse Transcription system (Promega). Quantitative PCR was performed with GoTaq qPCR Master Mix (Promega) using Universal Probe Library (Roche) or Taqman probes (Thermo Fisher Scientific) for detection. Primer sequences and Taqman probes are listed in Tables S2 and S3. GraphPad Prism software was used for graphic representation. Each analysis was performed in parallel on separate cell lines and data are presented as means from technical duplicates for each line.

### Flow cytometry

Cells were dissociated using Accutase (Innovative Cell Technologies) and washed using PBS with 2% foetal calf serum (FCS). For surface marker staining, cells were incubated with directly conjugated antibodies (Table S4) diluted in PBS with 2% FCS for 30 min at 4°C, followed by washing and resuspending in PBS. For intracellular marker staining, the cells were fixed with Fixation Buffer (00-8222-49, Thermo Fisher Scientific) for 30 min at 4°C, washed with Permeabilization Buffer (00-8333-56, Thermo Fisher Scientific) and incubated with antibodies diluted with Permeabilization Buffer and 5% donkey serum (Sigma-Aldrich) for 1 h at 4°C. Detection was performed using a BD LSRFortessa cell analyser (BD Biosciences), using FlowJo software for analysis.

### Immunofluorescence

Fixation was performed with 4% formaldehyde in PBS for 15 min, permeabilisation with 0.5% Triton X-100 in PBS for 10 min, blocking with 3% BSA and 0.1% Tween-20 in PBS for 30 min, all at room temperature. Antibodies (Table S5) were diluted in PBS with 0.1% Triton X-100 and 3% donkey serum. Incubation with primary antibodies was carried out overnight at 4°C, and then secondary antibodies were added for 1 h at room temperature. Slides were mounted using Prolong Diamond Antifade Mountant (Life Technologies, P36970).

### Assessment of mitochondrial membrane potential

Tetramethylrhodamine, methyl ester (TMRM) was applied to cells in culture medium at 100 nM for 30 min at 37°C. Fluorescence was measured using flow cytometry, then an uncoupling agent of mitochondrial oxidative phosphorylation FCCP (carbonyl cyanide-p-trifluoromethoxyphenylhydrazone) was added to cell suspension at 1 µM for 5 min and the measurement was repeated.

### Microscopy, image processing and quantification

Immunofluorescent imaging was performed on a Leica DMI4000 microscope or Andor Revolution XD spinning disk system for myotube visualisation. Immunofluorescent images of myotubes were deconvolved using Autoquant X3 (MediaCybernetics) and maximum intensity projections created using Imaris (Bitplane). For quantification of immunofluorescent staining for KLF17, NANOG, KLF4, OCT4, mean intensity of staining over identified DAPI nuclei was measured using Cell Profiler (Broad Institute). Movies were acquired using a Leica DMI4000 microscope, saved as .avi using Fiji software and then converted to .mp4 format.

### Library preparation and RNA sequencing

Total RNA was prepared using Reliaprep RNA Miniprep kit (Promega). Ribosomal RNA was depleted using Illumina's Ribozero HMR kit, according to the manufacturer's instructions. Libraries were prepared using the KAPA Stranded mRNA-Seq Kit (Kapa Biosystems, Roche) on an Agilent Bravo liquid handling system and MJ thermocyclers. Libraries were sequenced on HiSeq 2500, single end 50 bp reads.

### Transcriptome analysis

Reads were aligned to human genome build GRCh38/hg38 with STAR 2.5.2b (Dobin et al., 2013) and human gene annotation from Ensembl release 87 (Yates et al., 2016). Htseq-count (Anders et al., 2015) was used to quantify expression to gene loci. Mouse samples were compiled from an earlier study (Mohammed et al., 2017) and analysed as described in Boroviak et al. (2018). The Macaque FKPM expression dataset was provided by the authors (Nakamura et al., 2016). Orthology 1-to-1 for cross-species comparison was used.

Cluster analysis and principal component analysis were computed using log2 FPKM values with the Bioconductor packages DESeq2 (Love et al., 2014) or FactoMineR (Lê et al., 2008) in addition to custom scripts. SCDE R package (Kharchenko et al., 2014) was used to perform differential expression analysis. Fractional identity between the sample of the time-course and the selected embryo stages was determined via quadratic programming using the R package DeconRNASeq (Gong and Szustakowski, 2013). Average expression levels of the cells that comprise distinct macaque embryo stages were used as the ‘signature’ dataset, and the relative identity of each time-course sample was computed by quadratic programming. Enrichment of KEGG pathways was computed with custom script using the KEGG database resource (https://www.genome.jp/kegg/). Soft clusters were computed with R package MFuzz (Futschik and Carlisle, 2005; Kumar and Futschik, 2007) and the elbow method was used to determine the appropriate number of clusters. In order to detect genes with the greatest expression variability, a non-linear regression curve was fitted between log2 FPKM expression and the square of the coefficient of variation. Thresholds were applied along the *x*-axis (log2FPKM) and *y*-axis (logCV2) to identify the most variable genes.

## Supplementary Material

Supplementary information
