# Supplementary material for: Capacitation of human naïve pluripotent stem cells for multi-lineage differentiation
Source: Development. 2019 Apr 3;146(7):dev172916. doi: 10.1242/dev.172916 (PMC6467473; doi:10.1242/dev.172916)
Supplement: Supplementary information [file develop-146-172916-s1.pdf]

## SUPPLEMENTARY INFORMATION

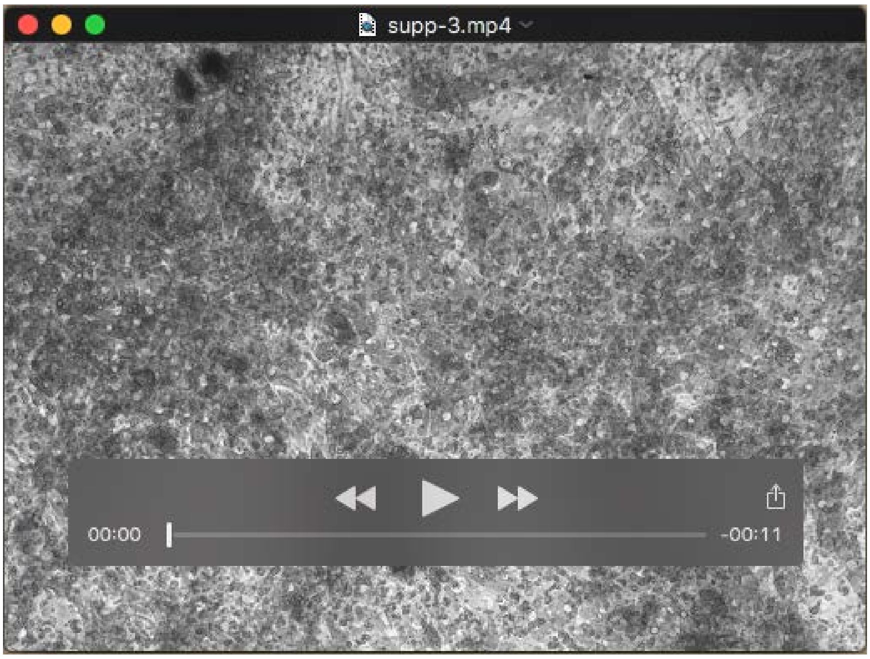


**Movie 1.** Reset cR-H9-EOS cells after formative transition and differentiation to myotubes for 40 days.

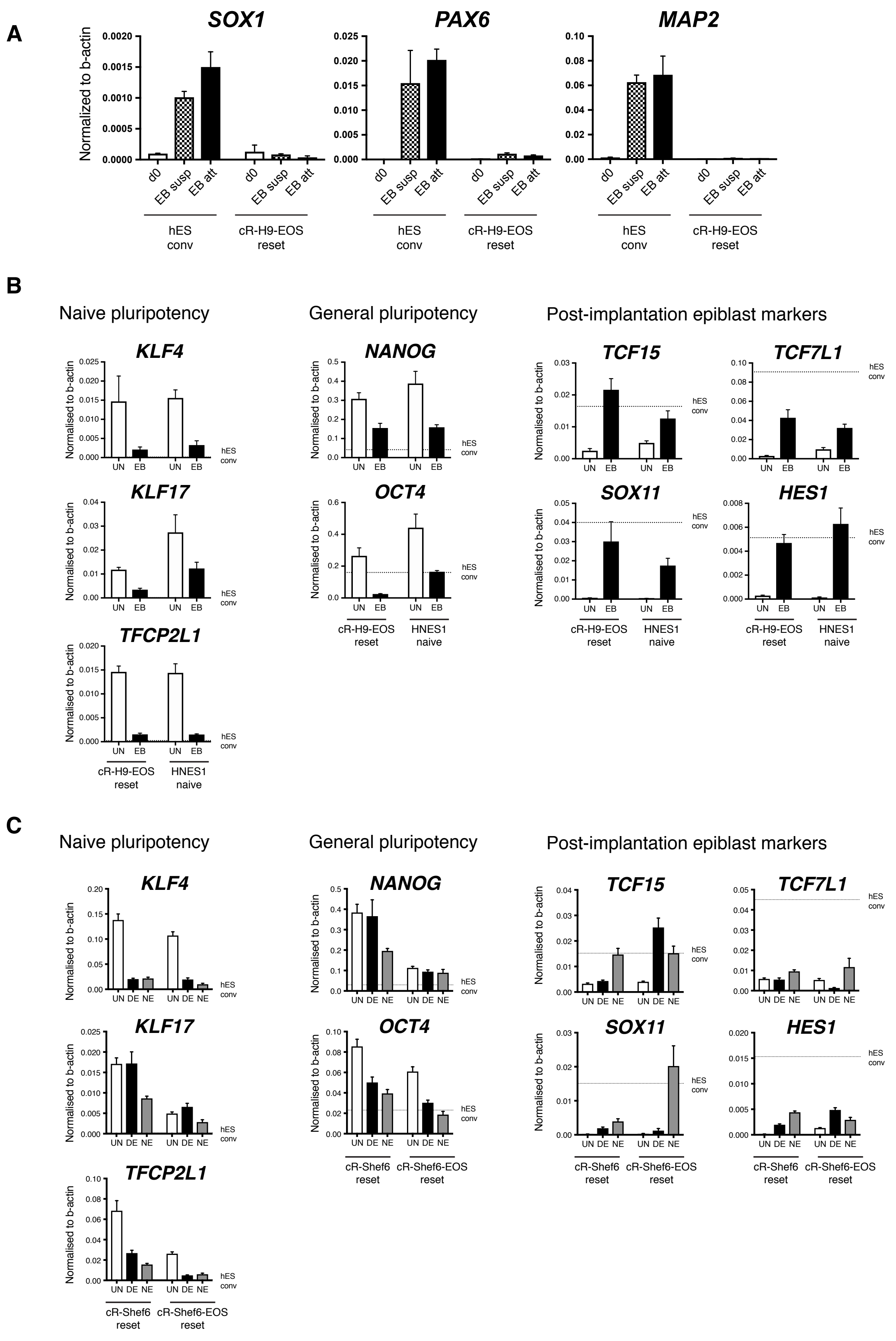


**Figure S1. Human naïve PSC do not respond to somatic differentiation protocols.** (A) Neuroectodermal markers are not induced directly from the naïve state. RT-qPCR analysis of neuroectodermal markers in aggregates cultured in suspension for 14 days (susp) or plated after 7 days in suspension and outgrown in adherent culture until day 14 (att). (B, C) Expression of markers after human naïve PSC were exposed to differentiation conditions: (B) embryoid bodies for 14 days, (C) monolayer

Development • Supplementary information

induction of definitive endoderm for 3 days and neuroectoderm for 10 days. Expression level in conventional hESC is indicated as dotted line.
UN – undifferentiated, EB – embryoid bodies, DE – definitive endoderm, NE – neuroectoderm.
The data in Fig. S1B, S1C are derived from experiments also presented in Figure 1B, 1D, 1F. Expression of NANOG and OCT4 in Fig. S1B is the same as in Figure 1B.

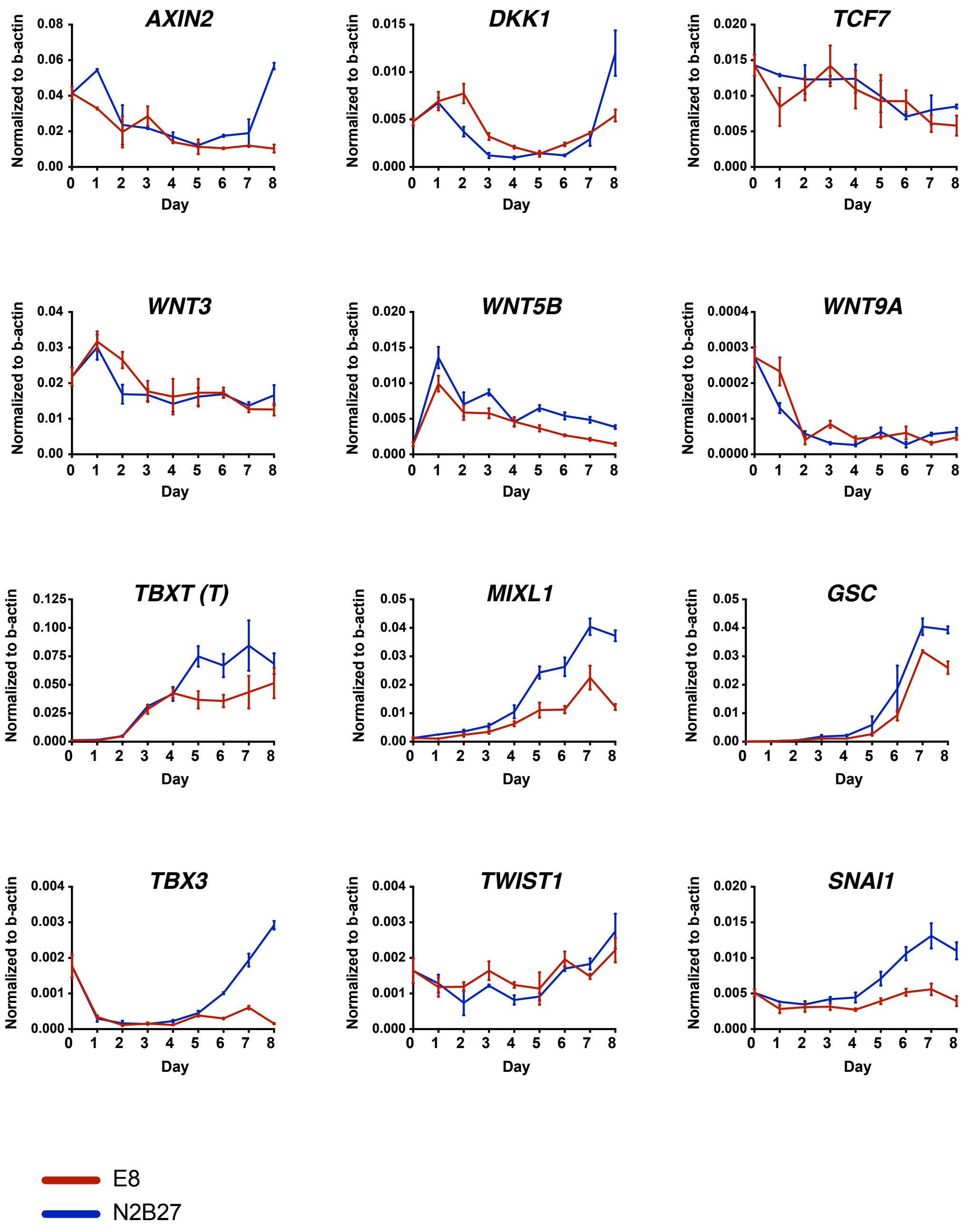


**Figure S2. Expression of WNT signalling pathway members and targets in N2B27 or E8**
RT-qPCR analysis

Development • Supplementary information

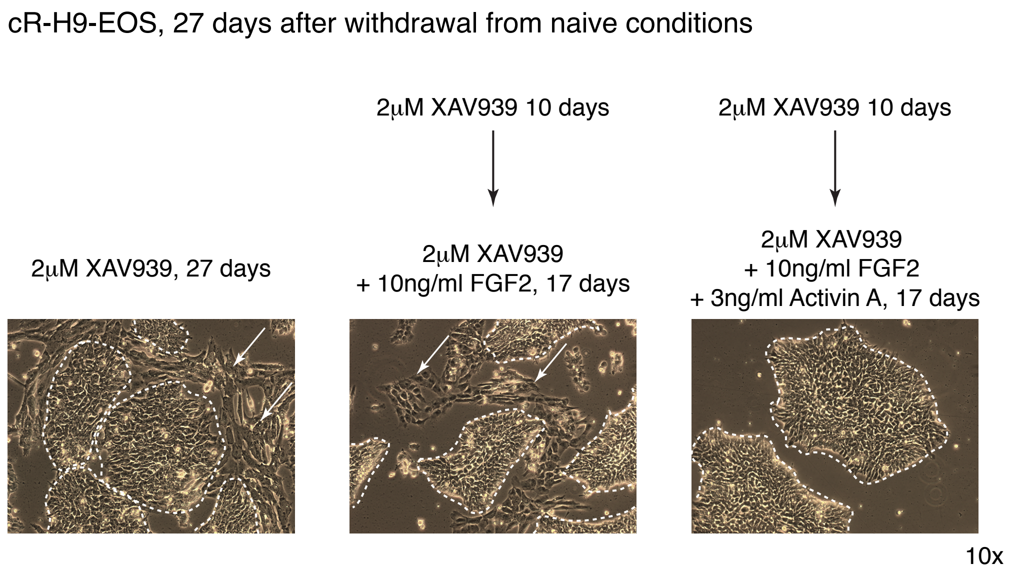


**Figure S3. hPSC can be maintained after formative transition**
Images of cR-H9EOS cells cultured after 10 days transition in XAV939 for 27 days in total in the indicated conditions, 10x. Arrows indicate spontaneously differentiated cells, dotted contours outline undifferentiated colonies.

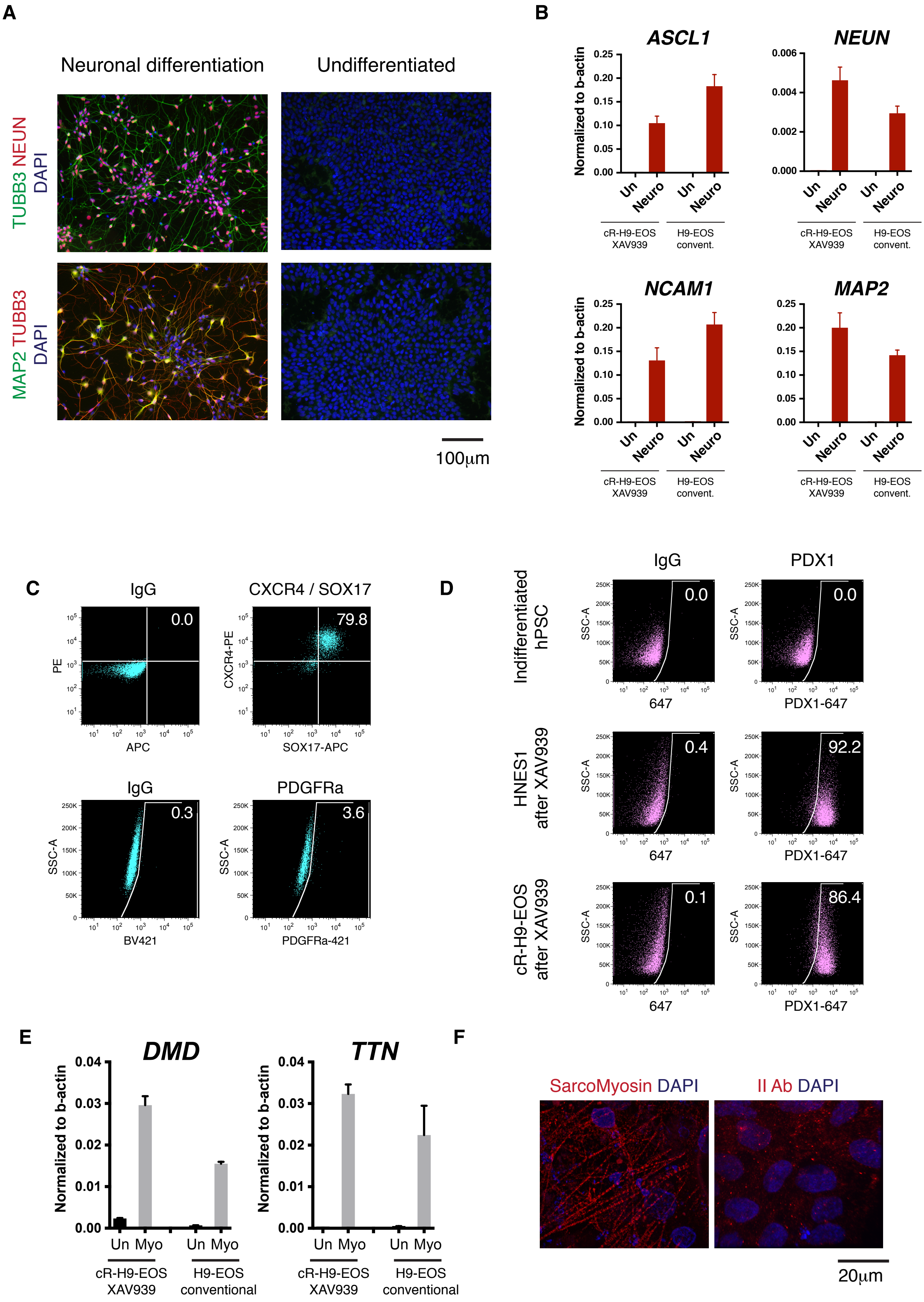


**Figure S4. Further differentiation after formative transition in XAV939.**

(A) Immunostaining for neuronal markers of capacitated cells differentiated to neurons and undifferentiated (capacitated cells which were not induced to differentiation). (B) RT-qPCR analysis of neuronal marker expression. (C) Flow cytometry analysis for CXCR4, SOX17 and PDGFRa upon induction of definitive endoderm following capacitation in XAV939. (D) Intracellular flow cytometry analysis for PDX1 in capacitated naïve hPSC subjected to definitive endoderm induction and subsequent differentiation to foregut endoderm and undifferentiated (capacitated cells which were not induced to differentiation). (E) Expression of sarcomeric markers in myotubes differentiated from capacitated naïve and conventional hPSC. (F) Immunostaining for sarcomeric myosin (MF20 antibody) in myotubes differentiated from capacitated naïve hPSC; control staining with secondary antibody only.

Un – undifferentiated, Neuro – neurons, Myo – myotubes.

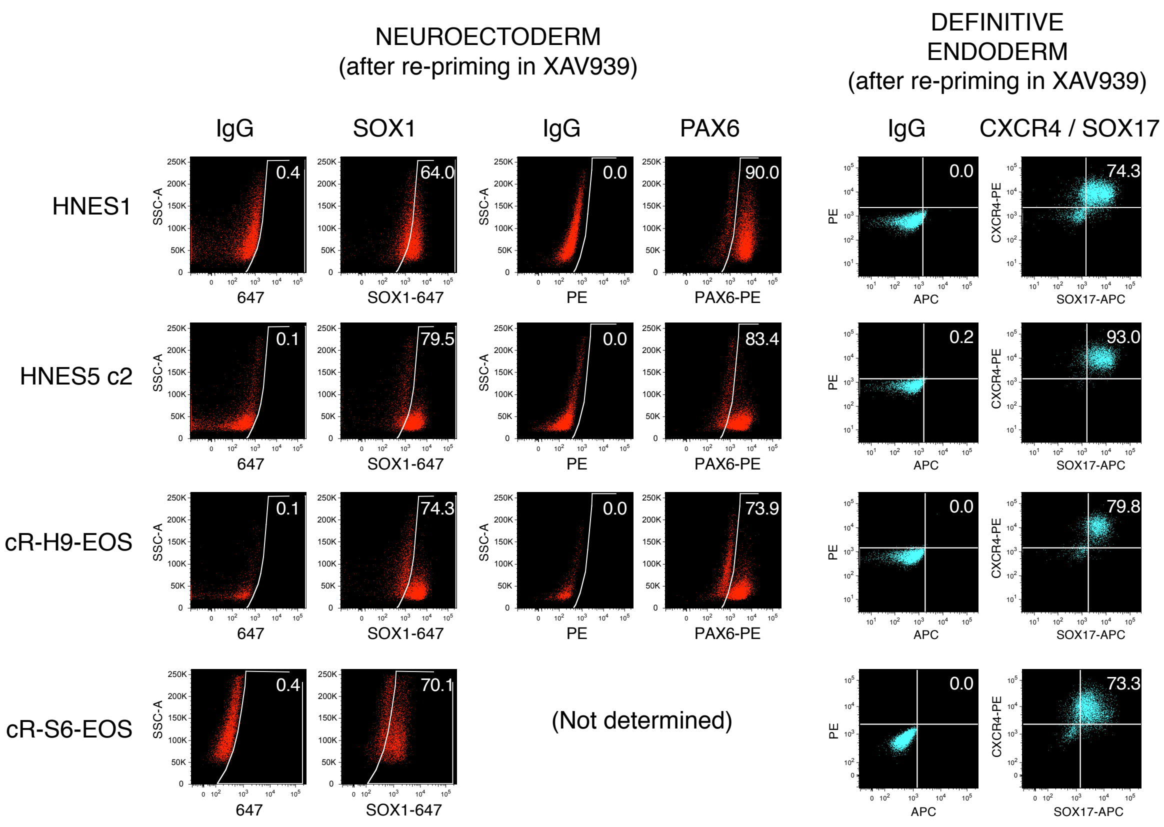


**Figure S5. Differentiation competence of various naïve hPSC after capacitation in XAV939.**

Neuroectoderm induction was analysed by flow cytometry for PAX6 and SOX1. Definitive endoderm induction was analysed by flow cytometry for CXCR4 and SOX17.

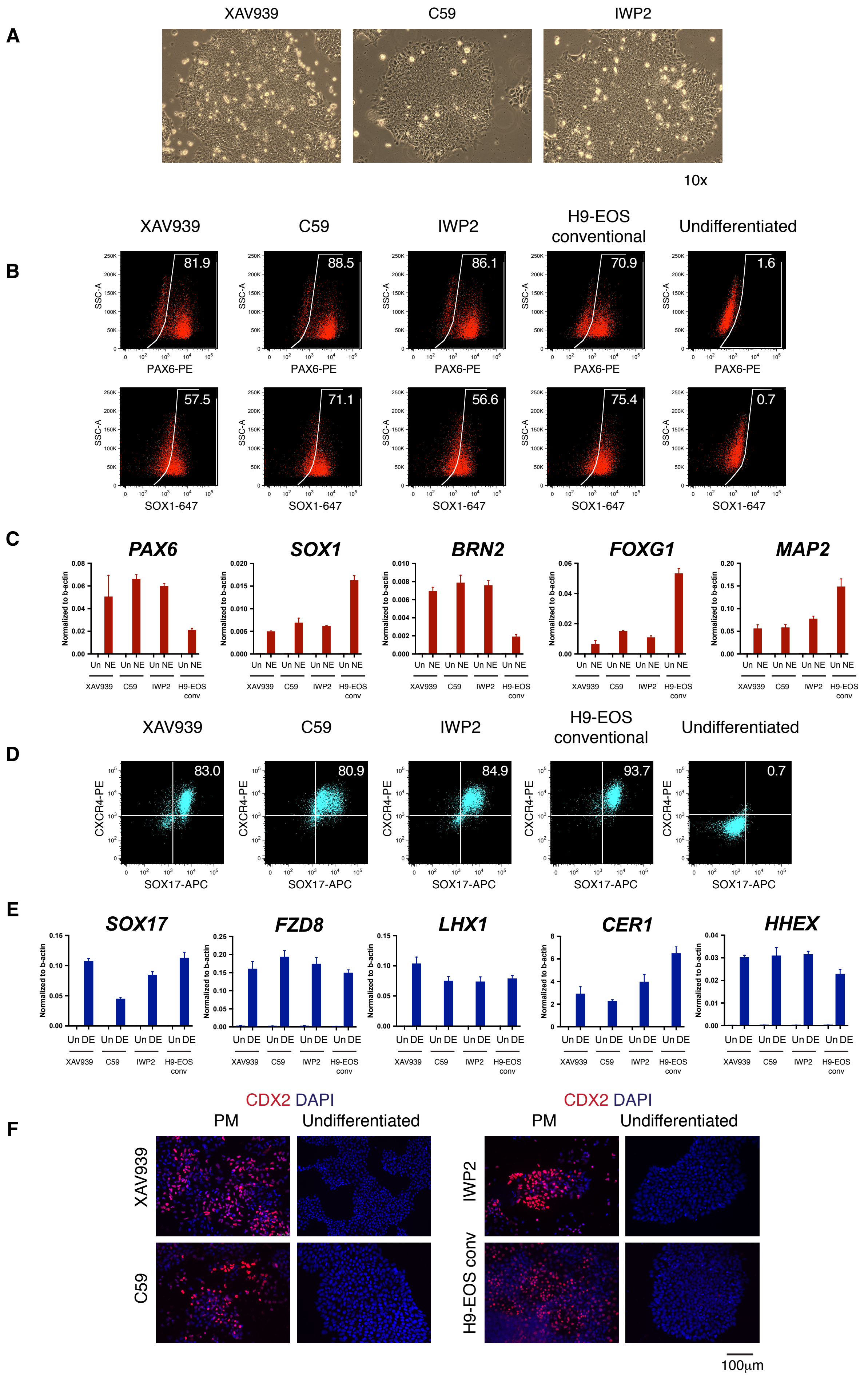


**Figure S6. Formative transition using different WNT pathway inhibitors.**

Development • Supplementary information

(A) Cell morphology after formative transition in XAV939, C59 or IWP2 for 12 days (bright field, 10x). (B,C) Neuroectoderm induction after capacitation in XAV939, C59 or IWP2, analysed by: (B) flow cytometry for PAX6 and SOX1; (C) RT-qPCR. (D,E) Definitive endoderm induction after capacitation in XAV939, C59, IWP2, assayed by: (D) flow cytometry for CXCR4 and SOX17; (E) RT-qPCR. (F) Immunostaining for CDX2 after paraxial mesoderm induction of cells capacitated in XAV939, C59, IWP2. Undifferentiated cells (capacitated cells which were not induced to differentiation) were used as a control in (B-F).

NE – neuroectoderm, DE – definitive endoderm, PM – paraxial mesoderm, Un – undifferentiated.

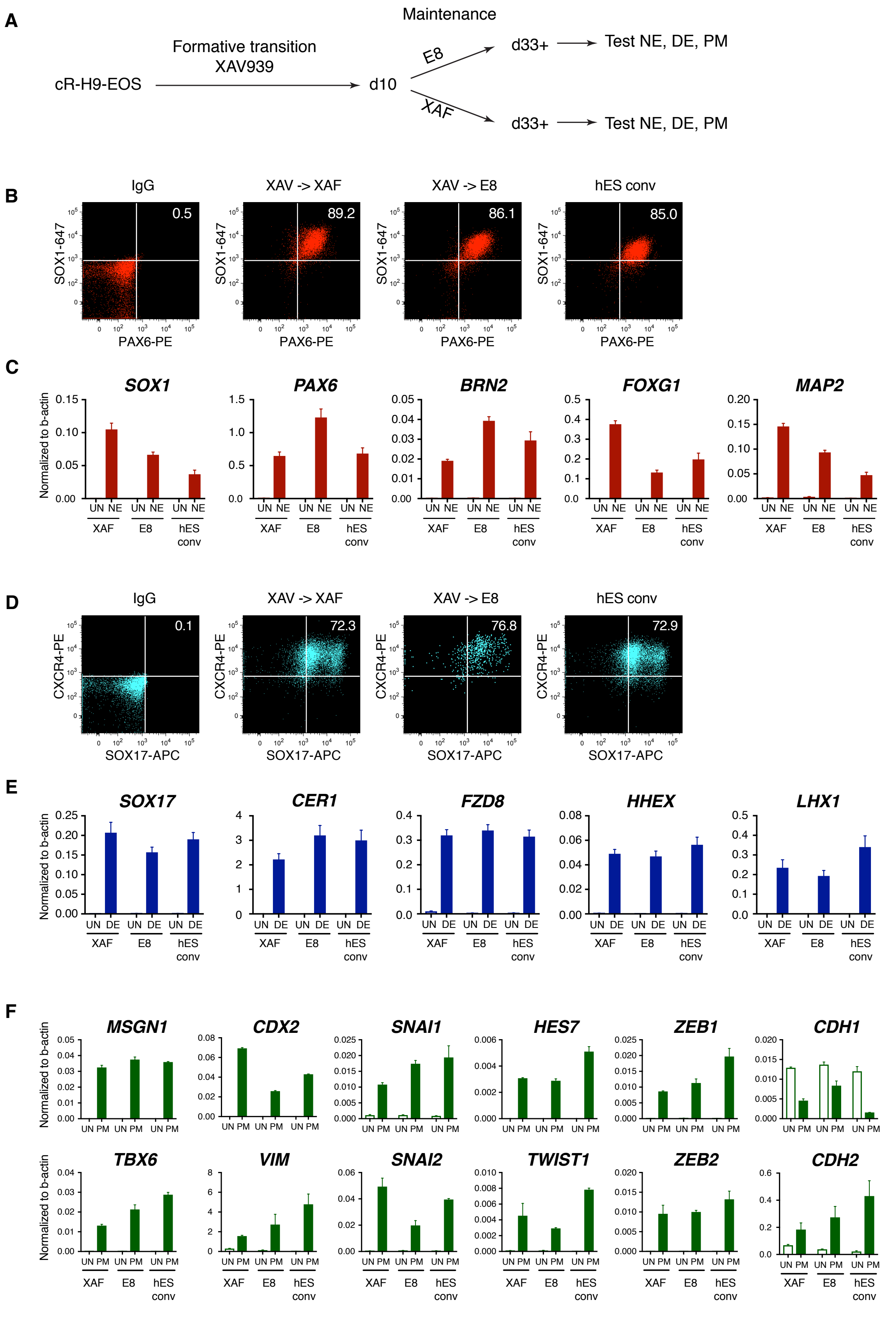


**Figure S7. Differentiation capacities of PSC after capacitation in XAV939 followed by extended maintenance.**
(A) Experimental setup. (B, C) Neuroectoderm differentiation analysed by: (B) flow cytometry for PAX6 and SOX1 and (C) RT-qPCR. (D,E) Definitive endoderm differentiation analysed by: (D) flow cytometry for SOX17 and CXCR4 and (E) RT-qPCR. (F) Paraxial mesoderm induction analysed by RT-qPCR.

Development • Supplementary information

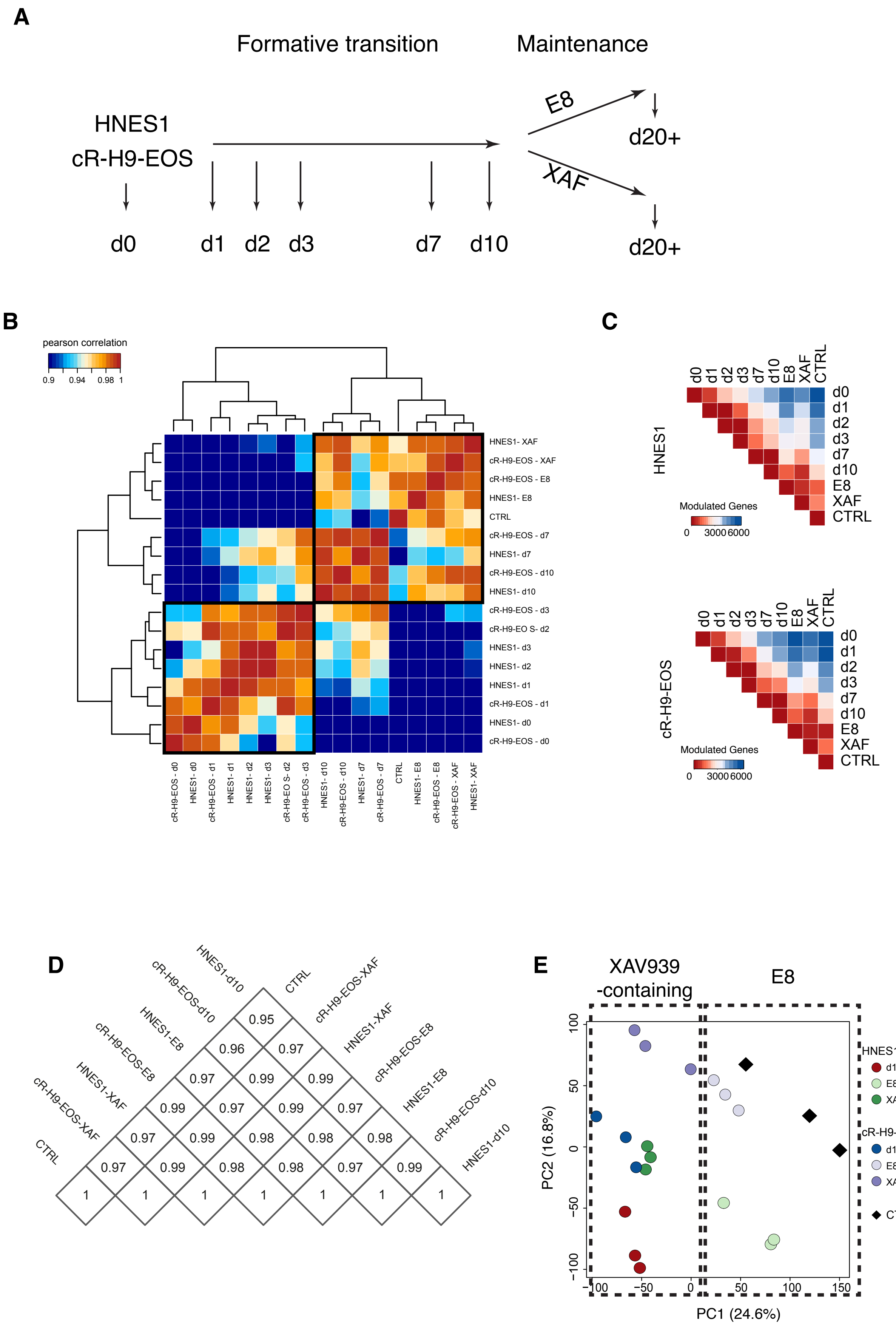


**Figure S8. Transcriptome profiling of naïve hPSC during formative transition and maintenance.**

Development • Supplementary information

(A) Experimental setup of global gene expression analysis during formative transition. (B) Heatmap showing correlations between samples during capacitation, after prolonged culturing, and with conventional H9EOS hPSC. Clusters of similar samples are outlined. (C) Heatmaps showing numbers of modulated genes in pair-wise comparisons between all points of the time course, CTRL – conventional H9-EOS. (D) Analysis of correlation coefficients after further culture in XAF or E8, and H9-EOS conventional cells and (E) PCA plot performed with all expressed genes.

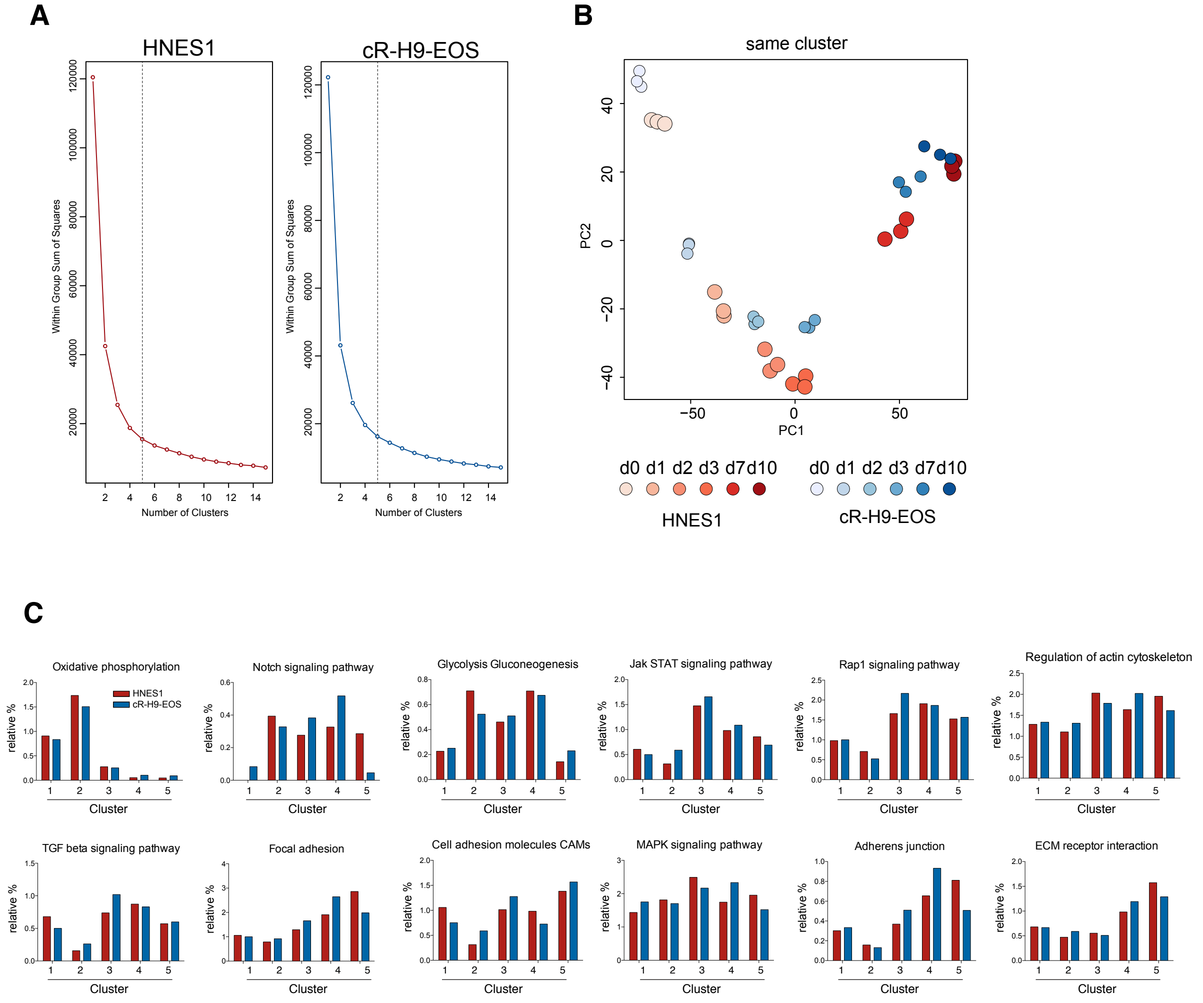


**Figure S9. Clustering analysis of variable genes during formative transition**
(A) Soft clustering analysis was performed for the set of genes variable between any two time points during the transition and in at least one of the cell lines. Number of clusters was defined using elbow method. (B) PCA of samples during formative transition, performed with genes belonging to the same clusters in HNES1 and cR-H9EOS. (C) Representation of KEGG pathways within clusters of variable genes. The bars indicate proportions of genes of each cluster belonging to indicated pathways.

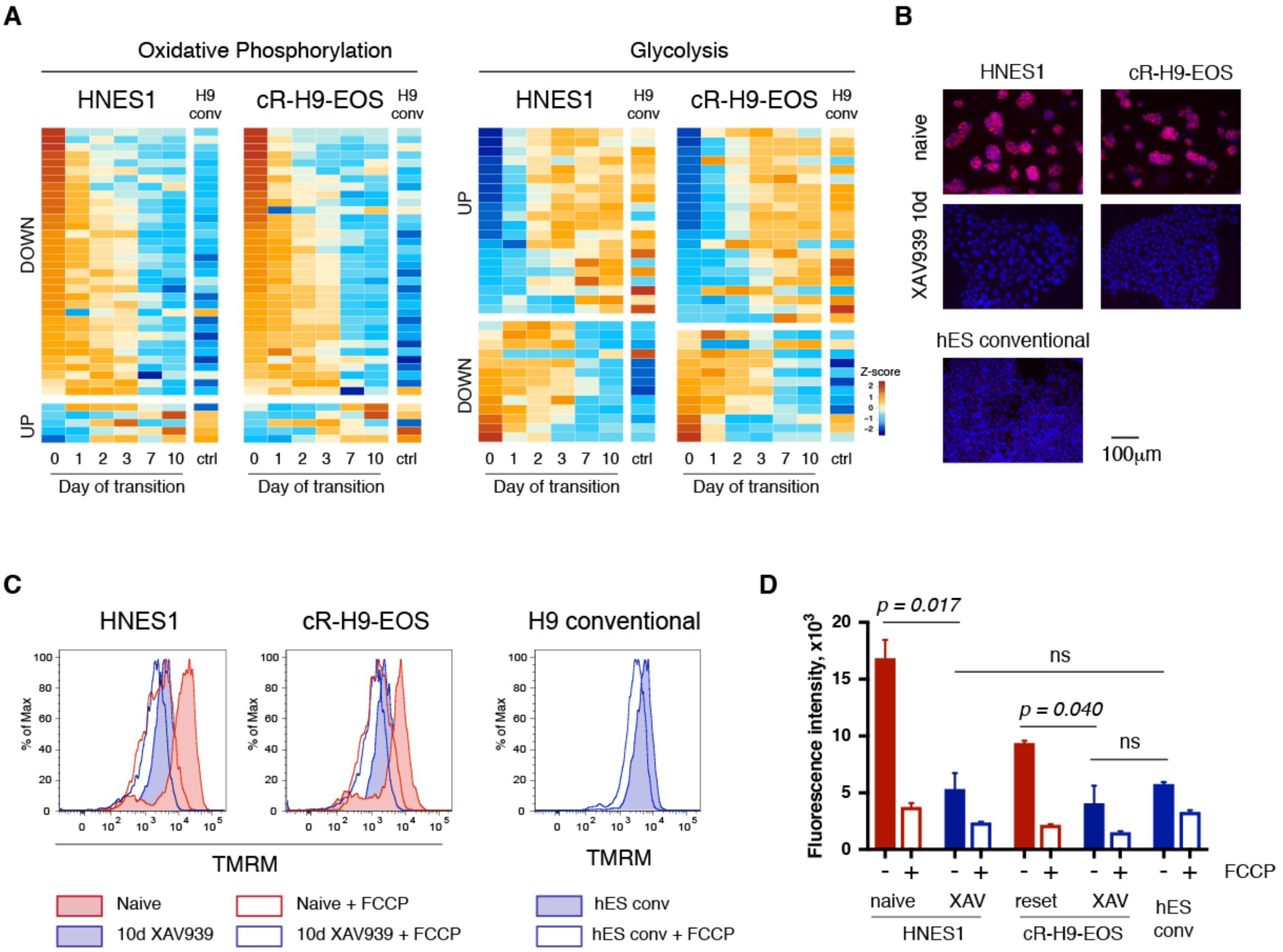


**Figure S10. Mitochondrial membrane potential reduces during formative transition.**
(A) Heatmaps showing genes involved in oxidative phosphorylation and glycolysis (according to KEGG pathways annotation), that are differentially expressed during the formative transition. The genes were ordered according to Z score. (B) Fluorescent images showing TMRM staining in naïve HNES1 and reset cR-H9-EOS before and after the formative transition, as compared to conventional hPSC. Counterstaining Hoechst33342. (C) Flow cytometry results of TMRM staining in naïve and reset PSC, capacitated PSC, conventional hPSC, measured in the presence or absence of FCCP (carbonyl cyanide-p-trifluoromethoxyphenylhydrazone), an uncoupling agent of mitochondrial oxidative phosphorylation. (D) Quantitation of flow cytometry results as in (C), shown as fluorescence intensity, the values were derived from independent formative transitions for each cell line. *P*-values were calculated using Student's two-tailed t-test.

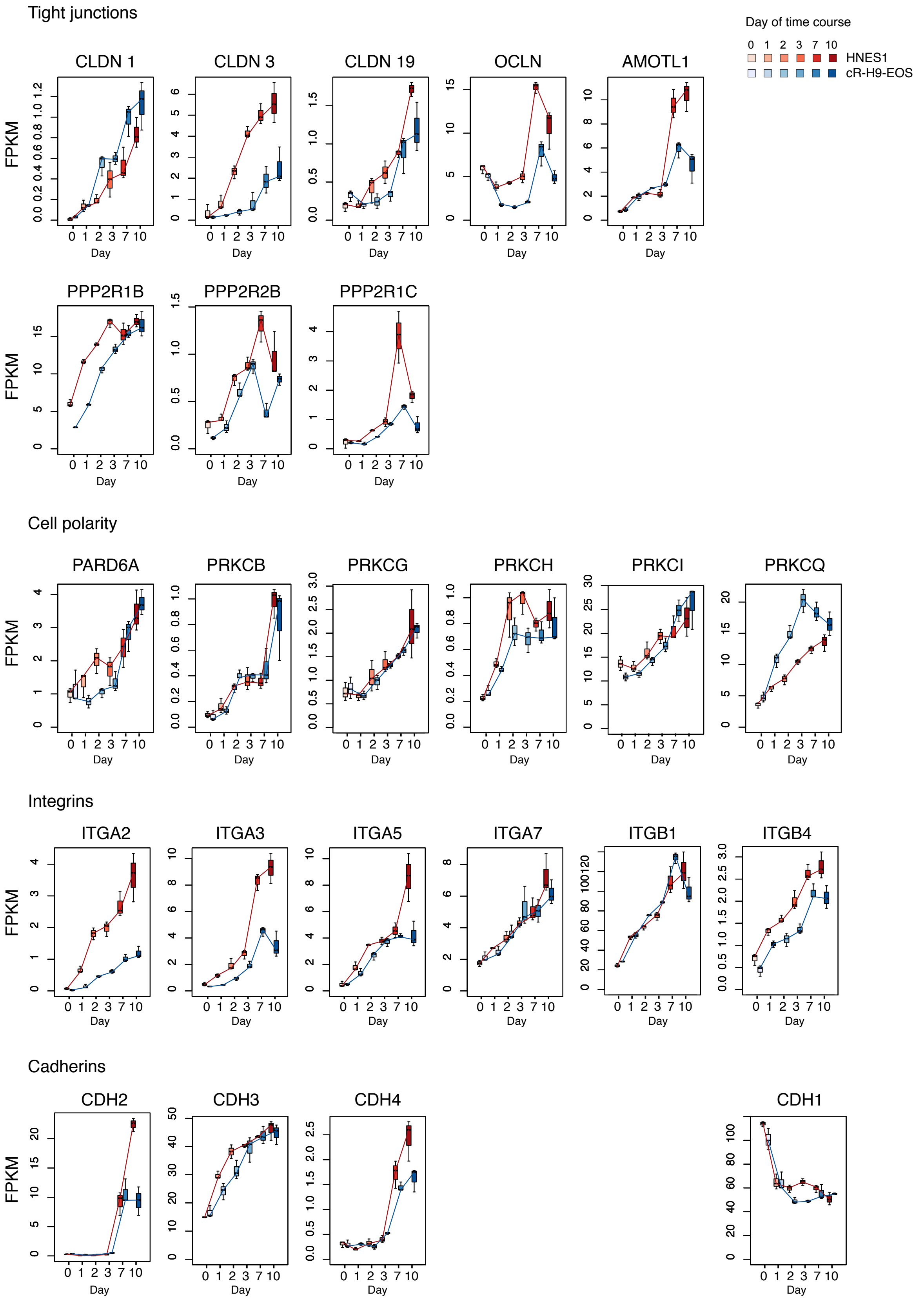


**Figure S11. RNAseq expression values for epithelial markers during the formative transition.**

Development • Supplementary information

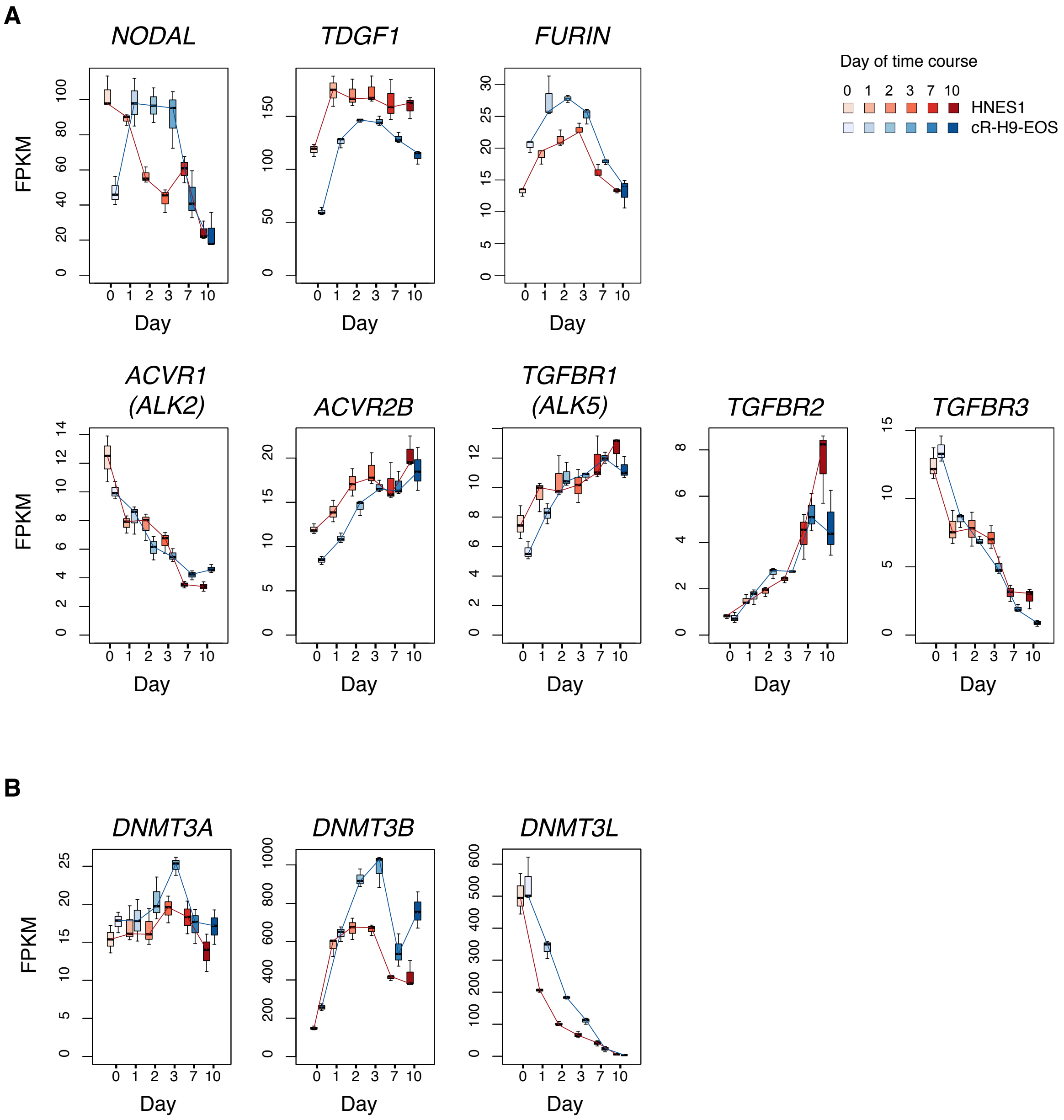


**Figure S12 Expression of selected genes during the formative transition**
(A) RNAseq expression values for genes associated with TGFb pathway. *INHBA*, *INHBB*, *TGFB1*, *PACE4*, *ACVR1B* (*ALK4*), *ACVR1C* (*ALK7*), *ACVR2A* showed very low or undetectable levels. (B) *DNMT3A*, *DNMT3B* and *DNMT3L* expression during formative transition.

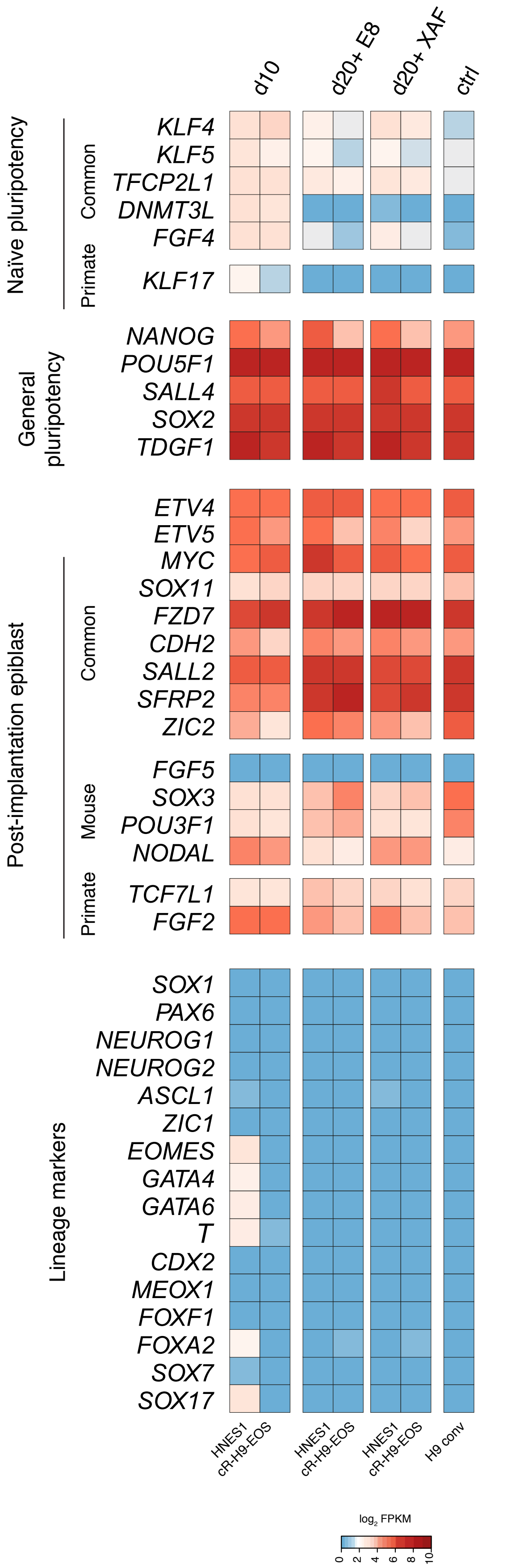


**Figure S13. Expression of selected genes in PSC after 10 days of capacitation in XAV939, after further maintenance in E8 or XAF, and conventional H9 hES.**

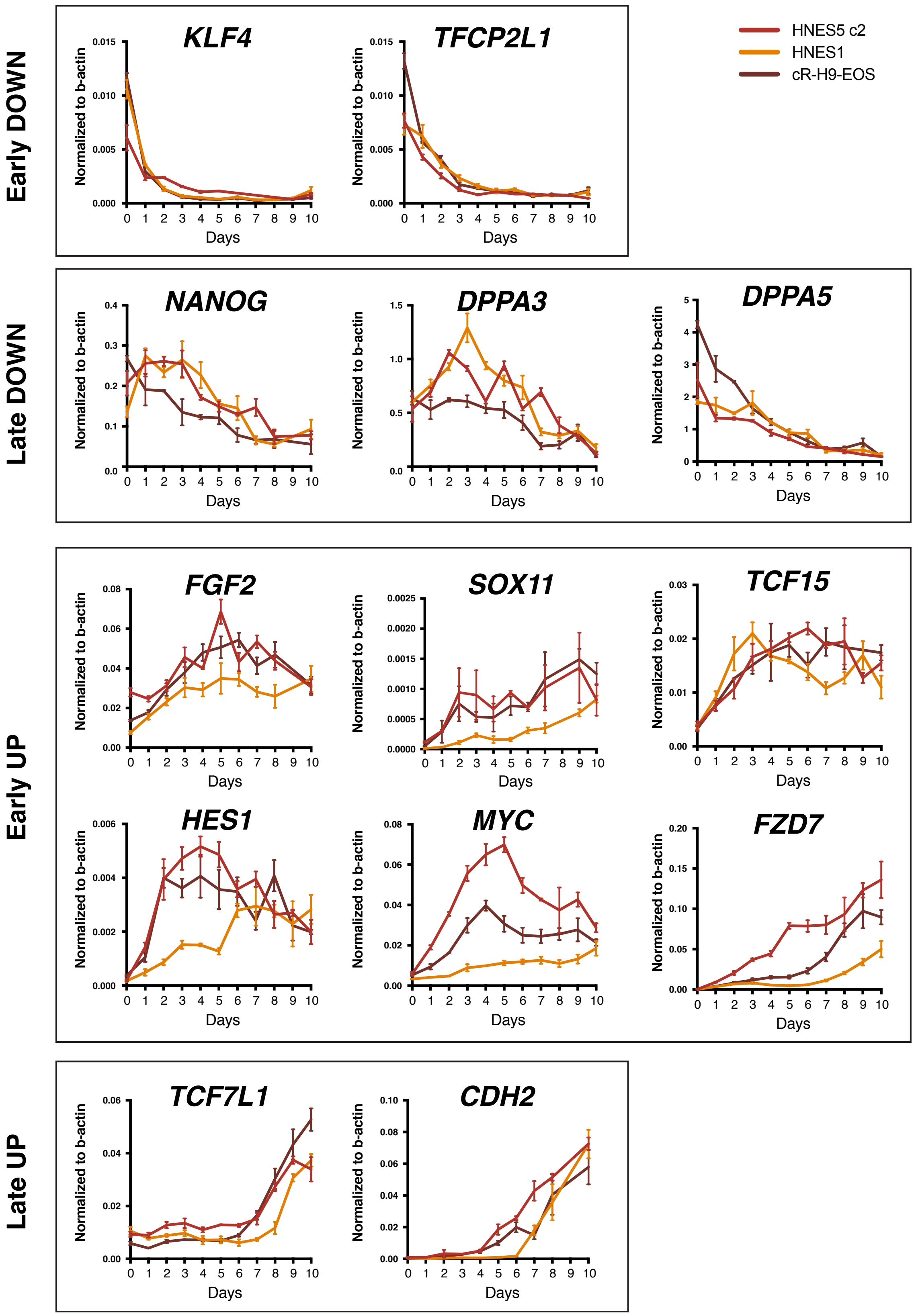


**Figure S14. Confirmation of selected gene expression dynamics during formative transition**

Expression of genes with different dynamics according to RNAseq was evaluated by RT-qPCR in an independent experiment using HNES1, HNES5c2 and cR-H9-EOS.

**Table S1. Summary of capacitation experiments on different cell lines**

| Naïve cell line | Parental cell line | WNT inhibitor | No. of independent experiments | Differentiation tests after capacitation |
|---|---|---|---|---|
| Embryo-derived HNES | | | | |
| HNES1 | N/A | XAV939 | 14 | NE, DE, PM |
| | | C59 | 2 | NE, DE, PM |
| | | IWP2 | 1 | NE, DE, PM |
| HNES5c1 | N/A | XAV939 | 1 | DE |
| HNES5c2 | N/A | XAV939 | 5 | NE, DE |
| Reset hESC | | | | |
| cR-S6-EOS-vpc | Shef6-EOS* | XAV939 | 1 | NE, DE |
| cR-S6-EOS-vpcx | Shef6-EOS* | XAV939 | 1 | NE, DE |
| cR-S6-EOS-vpcy | Shef6-EOS* | XAV939 | 1 | NE, DE |
| cR-H9-EOS-PC25B | H9-EOS-1* | XAV939 | 2 | NE, DE |
| cR-H9-EOS-vpc | H9-EOS-2* | XAV939 | 1 | NE, DE |
| cR-H9-EOS-vpcx | H9-EOS-2* | XAV939 | 11 | NE, DE, PM |
| | | C59 | 2 | NE, DE, PM |
| | | IWP2 | 2 | NE, DE, PM |
| Reset hiPSC | | | | |
| cR-LQT1 #26 | LQT1 #26 | XAV939 | 1 | - |
| cR-LQT1 #26-9.3-C6 | LQT1 #26-9.3-C6 | XAV939 | 1 | DE |

* chemically reset lines derived from the same parental cell line in independent resetting experiments are shown separately
N/A – not applicable

NE – neuroectoderm, DE – definitive endoderm, PM – paraxial mesoderm

Development • Supplementary information

**Table S2. List of primers.**

| mRNA | Forward (5'-3') | Reverse (5'-3') | Product size | UPL probe |
|---|---|---|---|---|
| *OCT4* | cttcgcaagccctcatttc | gagaaggcgaaatccgaag | 88 | 66 |
| *NANOG* | agatgcctcacacggagact | tttgcgacactcttctctgc | 127 | 31 |
| *SOX2* | tgctgcctctttaagactaggac | cctggggctcaaacttctct | 75 | 35 |
| *KLF4* | gggagaagacactgcgtca | ggaagcactgggggaagt | 88 | 52 |
| *TFCP2L1* | gctcttcaacgccatcaaa | caggggcactcgattctg | 88 | 17 |
| *KLF17* | ctcctgctgctggtccttag | acagttgccacgtccagtg | 74 | 64 |
| *DPPA3* | aatgctagaatagggaatcaagaca | agcatagagtagctttctcaacctg | 114 | 41 |
| *AXIN2* | tctgtggggaagaaattccata | caaactcatcgcttgcttttt | 129 | 88 |
| *DKK1* | ttctccctcttgagtccttctg | ctaccatcgcgacaaagacc | 76 | 21 |
| *TCF7* | ccctgacctctctggcttct | tcaaggatgggtgggtga | 89 | 27 |
| *WNT3* | cctgcaagtagggcacca | cccatgagacttcgctgaat | 74 | 5 |
| *WNT5B* | ctgctgctgctgttcacg | ccgggttcaaagctaatgac | 91 | 56 |
| *WNT9A* | cgtcttgtgggtgtcctga | cctgtatccataccagcaacc | 64 | 23 |
| *MIXL1* | ggtaccccgacatccactt | gcctgttctggaaccatacct | 87 | 32 |
| *GSC* | cctccgcgaggagaaagt | cgttctccgactcctctgat | 92 | 29 |
| *TBX3* | gcagctttcaactgcttcg | accctcgctgggacataaat | 90 | 19 |
| *TWIST* | gggccggagacctagatg | tttccaagaaaatctttggcata | 112 | 50 |
| *TCF7L1* | ccatgaacgcctcgatgt | gagccaccatgtgaggaga | 60 | 54 |
| *FGF2* | cacttcaaggaccccaagc | cctctctcttctgcttgaagttg | 137 | 4 |
| *SOX11* | tccgtgctggtgattttatg | acgttgccaacaagtttcg | 61 | 84 |
| *TCF15* | tgttccgggacactctgg | caggctgaatggatcctcac | 78 | 80 |
| *HES1* | gtgaagcacctccggaac | gtcacctcgttcatgcactc | 113 | 60 |
| *MYC* | tgctccatgaggagacacc | cttttccacagaaacaacatcg | 92 | 77 |
| *FZD7* | gccagcttgtgcctaatagaa | agccgggagaaactcacag | 61 | 54 |
| *SOX17* | acgccgagttgagcaaga | tctgcctcctccacgaag | 82 | 61 |
| *HHEX* | cggacggtgaacgactaca | agaaggggctccagagtagag | 76 | 61 |
| *LHX1* | atgcaacctgaccgagaagt | caggtcgctaggggagatg | 121 | 80 |
| *CER1* | gccatgaagtacattgggaga | cacagccttcgtgggttatag | 69 | 41 |
| *FZD8* | cgccacgcgttaatttct | ccggttctggaaccacac | 135 | 19 |
| *SOX1* | accaggccatggatgaag | cttaattgctggggaattgg | 67 | 37 |
| *PAX6* | ggcacacacacattaacacactt | ggtgtgtgagagcaattctcag | 71 | 9 |
| *BRN2* | aataaggcaaaaggaaagcaact | caaaacacatcattacacctgct | 72 | 57 |

Development • Supplementary information

| | | | | |
|---|---|---|---|---|
| *ASCL1* | gctcttacgacccgctca | atgcaggttgtgcgatca | 129 | 15 |
| *MAP2* | actgcagctctgcctttagc | gacagtctgttctgaggcaggt | 84 | 16 |
| *NEUN* | ccaccattttcccaggtct | attttccccgaggcactct | 96 | 1 |
| *NCAM1* | cgaccatccacctcaaagtc | cggaggcttcacaggtaaga | 105 | 68 |
| *FOXG1* | atgatccccaagtcctcgtt | gtggtggttgtcgttctgg | 69 | 64 |
| *VIM* | tgagattgccacctacaggaa | gagggagtgaatccagattagttt | 113 | 11 |
| *TBX6* | gaacggcagaaactgtaagagg | gtgtgtctccgctcccatag | 101 | 5 |
| *MSGN1* | agctcaggatgaggaccttg | ctggcctctctggctgtaga | 80 | 87 |
| *HES7* | gcagcctggaagagctga | acggcgaactccaatatctc | 100 | 78 |
| *CDH1* | ggtctgtcatggaaggtgct | gatggcggcattgtaggt | 95 | 5 |
| *CDH2* | tgcacagatgtggacaggat | ccacaaacatcagcacaagg | 106 | 15 |
| *SNAI1* | gcgagctgcaggactctaat | cggtggggttgaggatct | 102 | 62 |
| *ZEB1* | agcacttaagaattcacagtggag | catttcttactgcttatgtgtgagc | 106 | 36 |
| *ZEB2* | cgcagtgacacagccattat | gttccaggtggcaggtca | 104 | 17 |
| *TBXT* | gctgtgacaggtacccaacc | catgcaggtgagttgtcagaa | 106 | 23 |
| *TTN* | ccaagttacccatggaaaggt | actcgggcctggtctactg | 84 | 41 |
| *DMD* | ctgcgtggatatgtgtctgaa | acacggatcctccctgttc | 69 | 66 |

**Table S3. Taqman probes (all from ThermoFisher Scientific)**

| | |
|---|---|
| KLF4 | Hs00358836_m1 |
| KLF17 | Hs00703004_s1 |
| TFCP2L1 | Hs00232708_m1 |
| DPPA5 | Hs00988349_g1 |
| NANOG | Hs04399610_g1 |
| ACTB | Hs01060665_g1 |
| GAPD (GAPDH) | 4352934E |

**Table S4. Antibodies for flow cytometry.**

| Antibody | Cat. Number | Company | Dilution |
|---|---|---|---|
| PE conjugated mouse IgG2a anti-human CD184 (CXCR4) | 555974 | BD Pharmingen | 1:25 |
| APC conjugated goat polyclonal anti-human SOX17 | IC1924A | RnD Bio-Techne | 1:100 |
| BV421 conjugated mouse IgG2a anti-human CD140a (PDGFRa) | 562799 | BD Biosciences | 1:100 |
| Alexa647 conjugated mouse IgG1 anti-human SOX1 | 562224 | BD Biosciences | 1:100 |
| PE conjugated mouse IgG2a anti-human PAX6 | 561552 | BD Biosciences | 1:100 |
| Mouse monoclonal IgG2a anti-human PDX1 | MAB2419 | RnD Bio-Techne | 1:1000 |

**Table S5. Antibodies for immunostaining.**

| Antibody | Cat. number | Company | Dilution |
|---|---|---|---|
| Polyclonal goat IgG anti-human HNF-3b/FOXA2 | AF2400 | RnD Bio-Techne | 1:50 |
| Polyclonal goat IgG anti-human SOX17 | AF1924 | RnD Bio-Techne | 1:100 |
| Polyclonal goat IgG anti-human SOX1 | AF3369 | RnD Bio-Techne | 1:100 |
| Polyclonal rabbit IgG anti-PAX6 | AB2237 | Merck Millipore | 1:500 |
| Polyclonal rabbit IgG anti-human MAP2 | 4542 | Cell Signaling Technology | 1:100 |
| Polyclonal rabbit IgG anti-human NEUN | ABN78 | Merck Millipore | 1:100 |
| Monoclonal mouse IgG2a anti-human β-Tubulin 3 (TUBB3) | T8578 | Sigma-Aldrich | 1:1000 |
| Polyclonal rabbit IgG anti-human CDX2 | 3977S | Cell Signaling Technology | 1:100 |
| Polyclonal rabbit IgG anti-human TBX6 | ab38883 | Abcam | 1:400 |
| Polyclonal rabbit IgG anti-human NANOG | ab21624 | Abcam | 1:100 |
| Monoclonal mouse IgG1 anti-human NANOG | 14-5768-82 | eBiosciences ThermoFisher Scientific | 1:300 |
| Polyclonal goat IgG anti-human/mouse Brachyury (TBXT) | AF2085 | RnD Bio-Techne | 1:250 |
| Monoclonal mouse IgG2b anti-human OCT4, C-10 | sc-5279 | Santa Cruz | 1:100 |
| Polyclonal rabbit IgG anti-human GKLF (KLF4), H-180 | sc-20691 | Santa Cruz | 1:300 |
| Polyclonal rabbit IgG anti-human KLF17 | HPA024629 | Atlas Antibodies | 1:300 |
| Monoclonal mouse IgG2b anti-sarcomere myosin | MF20 | DSHB | 1:10 |

**Table S6.**
Excel spreadsheet showing expression values for genes involved in glycolysis and oxidative phosphorylation, derived from KEGG annotation. Log FPKM values.

Click here to Download Table S6
